# Selective and scalable oxygenation of heteroatoms using the elements of nature: air, water, and light

**DOI:** 10.3762/bjoc.19.82

**Published:** 2023-07-31

**Authors:** Damiano Diprima, Hannes Gemoets, Stefano Bonciolini, Koen Van Aken

**Affiliations:** 1 Ecosynth, Industrielaan 12, 9800 Deinze, Belgium; 2 Flow Chemistry Group, Van ’t Hoff Institute for Molecular Sciences (HIMS), University of Amsterdam, Science Park 904, 1098 XH, Amsterdam, The Netherlands; 3 Creaflow, Industrielaan 12, 9800 Deinze, Belgium

**Keywords:** catalyst-free, flow chemistry, oxygen, photochemistry, sustainable oxidation

## Abstract

Sustainable oxidation protocols aim to provide an environmentally friendly and cost-effective method for the production of various chemicals and materials. The development of such protocols can lead to reduced energy consumption, fewer harmful byproducts, and increased efficiency in industrial processes. As such, this field of research is of great importance and interest to both academia and industry. This work showcases a sustainable and catalyst-free oxidation method for heteroatoms (e.g., S, P, and Se) using only air, water and light. An additional reaction pathway is proposed in which the incorporated oxygen on the heteroatoms originates from water. Furthermore, the addition of certain additives enhances productivity by affecting kinetics. The industrial potential is demonstrated by conveniently transferring the batch protocol to continuous flow using the HANU flow reactor, indicating scalability and improving safety.

## Introduction

**Scheme 1:**
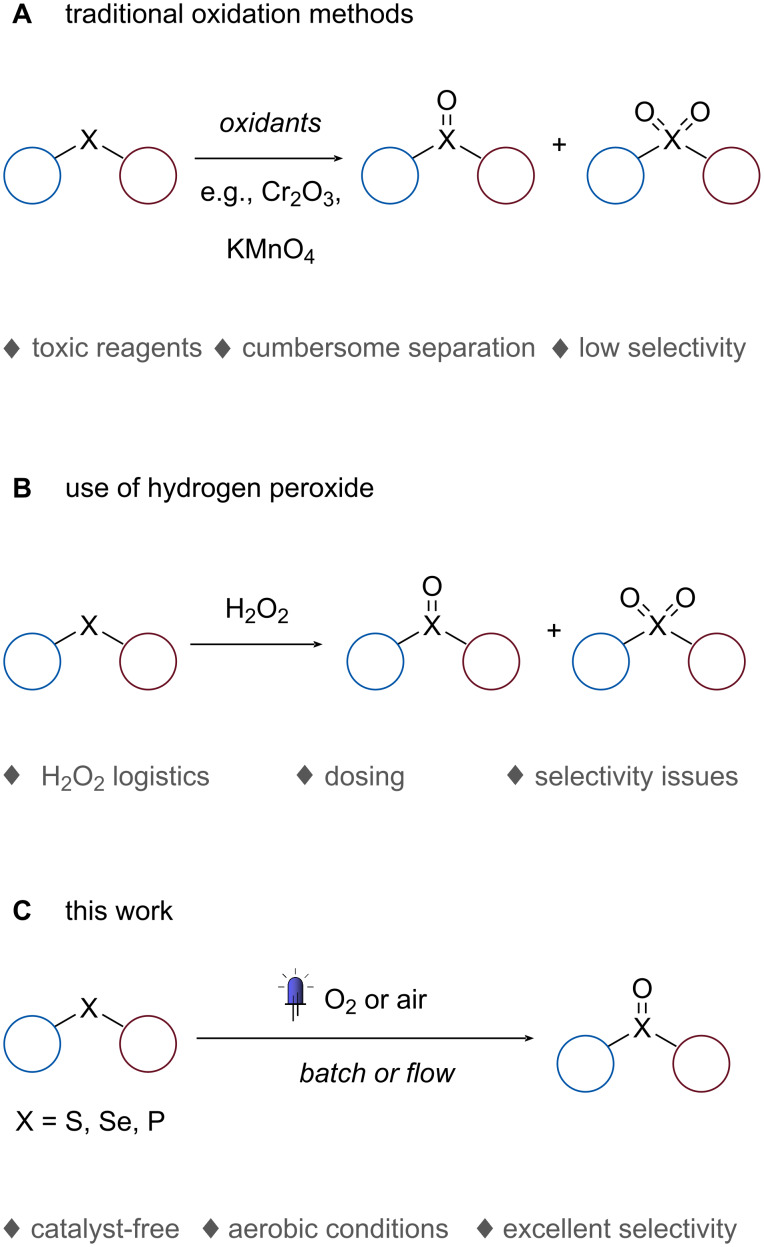
Oxidation of heteroatoms.

Oxidation reactions are widely used in the chemical industry, but are often problematic due to challenges with selectivity and safety. Traditional oxidants, such as Oxone, CrO_3_, NaIO_4_, or KMnO_4_, produce significant amounts of toxic waste, exacerbating these issues (Scheme 1A) [1]. As environmental concerns and economic factors increasingly affect chemical processes, hydrogen peroxide and oxygen (or air) are becoming more popular as oxidants due to their low cost and minimal side products. However, these reagents have practical limitations.

Hydrogen peroxide is typically produced off-site and requires transportation and storage, and is commonly obtained through the non-sustainable anthraquinone process (Scheme 1B) [2–4]. Additionally, practical implementation of hydrogen peroxide can be challenging due to requirements for precise dosing to avoid issues such as dismutation, overoxidation, and catalyst degradation [5]. In this respect, oxygen, or preferably air, represents a better alternative to traditional oxidants, but gas–liquid mass transfer limitations can reduce productivity. Additionally, in-depth safety studies are necessary to avoid the risk of explosion in batch reactors where an oxygen-rich head space is present along with flammable organic solvents.

These risks can be significantly mitigated by running the process in continuous flow [5–8]. Moreover, flow reactors that provide intense mixing can overcome the gas–liquid mass transfer limitations typical of batch reactions and improve productivity. Several studies have demonstrated the scalability and safety of such methods for the oxidation of heteroatoms, making them a promising alternative to traditional oxidants in the chemical industry [9].

Our interest in sustainable oxidation methodologies led us to study the selective oxidation of various heteroatoms to their corresponding oxides, including sulfides to sulfoxides, phosphine to phosphine oxide, and selenides to selenoxides. Sulfoxide, phosphine oxide, and selenoxide-containing molecules have diverse applications in the pharmaceutical industry [10], as chiral auxiliaries or as ligands for asymmetric metal catalysis [11], and in materials such as polymers [12–13] and flame retardants [14]. Sulfoxides are prominent pharmaceutical ingredients, while phosphine oxides improve solubility of corresponding compounds [15] and have applications in catalysis and materials science [16]. Selenoxides find use as oxygen transfer agents and donor ligands in metal catalysis and organic synthesis [17–20], although they are less commonly utilized than the other two functional groups.

Over time, various synthetic protocols for oxygenation reactions have evolved. Initially, stoichiometric amounts of toxic oxidants were used, but now more sustainable oxidants such as H_2_O_2_ [21–22], O_2_ [23–24] and methods for oxidation such as photochemistry, or electrochemistry have been developed [2,25]. However, low selectivity and the need for appropriate catalysts that are stable, cost-effective, and easy to remove remain problematic. Recently, catalyst-free procedures using O_2_ or air have emerged [26], but they suffer from low selectivity or long reaction times [27–28], making them unsuitable for large-scale industrial production.

In this study (Scheme 1C), we introduce a photochemical, catalyst-free oxidation method for heteroatoms that is highly selective and suitable for industrial implementation. The protocol utilizes oxygen or air, a water-based solution, and UV-A irradiation at 365 nm. Scaling up such protocols has traditionally been difficult, as demonstrated by a previous oxidation of thioanisole on a 10 g scale, which had a long reaction time of 13 hours [29]. Advantageously, our protocol demonstrates excellent scalability, as we have successfully transferred it to the HANU flow reactor. This flow photoreactor is designed for multi-phase reactions (solid–liquid, gas–liquid) [30–32], and allows for seamless scale-up to production scale. Finally, by the use of specific “easy to separate” additives, a significant rate enhancement could be obtained with a positive impact on productivity rates.

## Results and Discussion

**Scheme 2:**
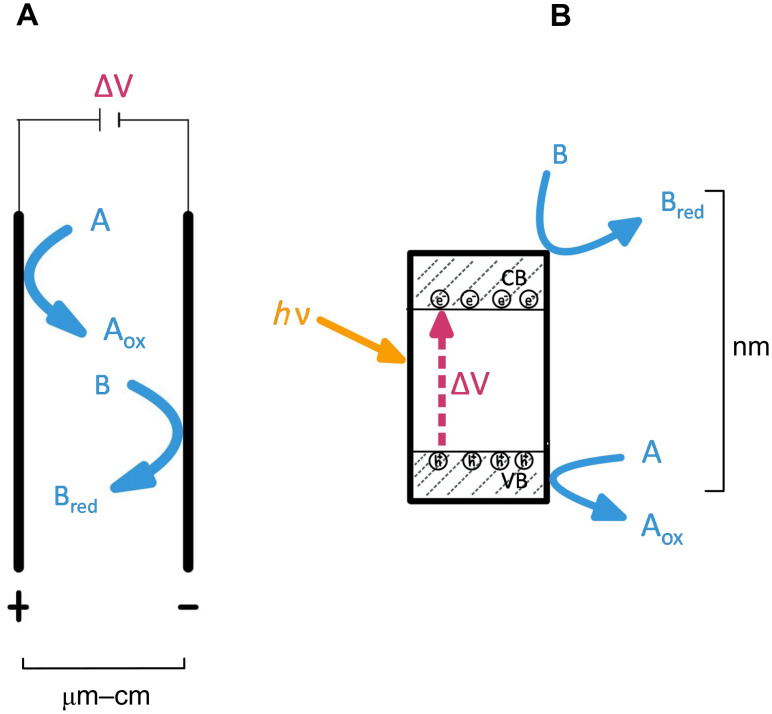
Graphical representation comparing A electrochemistry and B photoredox catalysis using a semiconductor photocatalyst.

There are a lot of similarities between electrochemistry and photoredox chemistry [33] as both rely on single-electron transfer processes to initiate reactions. In electrochemistry, the electron transfer occurs locally at the surface of the physical electrodes (typically located at a distance in the range of 200 μm to 2 cm) on which a potential is induced by an external potentiostat (Scheme 2). While for photoredox chemistry, the light-activated semiconductor catalyst behaves as a short-circuit electrochemical cell generating holes and free electrons at the particle surface at a much shorter distance from each other (in the range of nm, Scheme 2), which can lead to different chemical pathways and end products.

Interestingly, to the best of our knowledge, a benchmark comparative study between the electrochemical and the photoredox pathway, using the exact same chemical matrix, is not yet described. Intrigued by this, we decided to investigate the oxidation of sulfides both via electrochemistry and photoredox catalysis using thioanisole as benchmark substrate.

Initially, the optimized conditions from our group [34] from a previously reported electrochemical procedure [35] were employed (i.e., solvent CH_3_CN/H_2_O 80:20, 0.1 M *n*-Bu_4_Br, inert argon atmosphere). As for the photoredox catalyst we used the “first choice” TiO_2_ and irradiation with 365 nm LED light.

In the first run, a conversion of 10% was observed after 120 min irradiation time. Running the control experiments gave further insight into the critical components but also a few surprises. As expected, omitting the electrolyte in the photochemical procedure did not affect the conversion (so *n*-Bu_4_Br could be left out in future experiments) and no conversion was observed in the dark experiment. Surprisingly, after removal of the TiO_2_ photocatalyst from the reaction matrix, still an identical conversion of 10% was achieved. Additionally, replacing the inert atmosphere (argon) with oxygen had a drastic positive impact on the reaction outcome. By running the reaction in the presence of oxygen and in the absence of a photocatalyst, a quantitative conversion into the sulfoxide was obtained within 60 minutes in batch (Table 1, entry 1).

**Table 1:** Optimization experiments of thioanisole oxidation.^a^

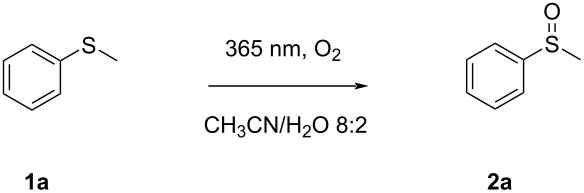				

Entry	Deviation	Time (min)	Conversion^b^ (%)	Selectivity^b^ (%)

1	none	60	>99	>99
2	H_2_O	40	97	98
3	CH_3_CN	60/120	47/97	97/80
4	EtOH/H_2_O	60	55	>99
5	bubbling air	60	99	>99
6	open vessel	60	99	>99
7	**1a** 0.6 M	70	94	>99
8	dark	120	0	
9	405 or 455 nm	120	0	

^a^The reaction is performed with **1a** (0.06 M) in 10 mL CH_3_CN/H_2_O 8:2 (v:v). The reaction is irradiated with a 365 nm 96 W LED lamp at 0.5 cm from the reactor wall. ^b^Based on GC-FID.

To shed more light on what could be the absorbing species within the reaction, and what would be the exact role of the present oxygen and water, we decided to conduct a series of comparative studies (Table 1). As a standard protocol, thioanisole (0.6 mmol) in 10 mL of solvent in a 22 mL test tube, equipped with a balloon containing O_2_, was irradiated using a UV-A LED (λ_max_ = 365 nm, 96 W) at a distance of 0.5 cm from the reactor wall.

In contrary with what has been reported previously for the catalyst-free oxidation under blue light irradiation [28], the reaction occurs also using water as the solvent (Table 1, entry 2). Running the reaction without adding extra water resulted in a significant reduction in kinetics and selectivity (Table 1, entry 3). Other more green and biobased solvent alternatives, such as ethanol [36], can effectively replace the acetonitrile (for the complete scope of solvents, please consult Supporting Information File 1, Table S2), but reaction rates were slower (Table 1, entry 4).

The oxygen excess appeared not to have an important impact as running the reaction using an open vessel or applying vigorous oxygen bubbling showed no significant difference on the overall reaction rate (Table 1, entries 1, 5, and 6).

Furthermore, it was possible to increase the concentration from 0.06 M to 0.6 M (Table 1, entry 7) maintaining approximately the same reaction time, while further concentration increase resulted in substantially slower kinetics (see Supporting Information File 1, Table S2).

When performing the reaction in the dark or under visible light irradiation (e.g., at 405 or 455 nm) no conversion was observed (Table 1, entries 8 and 9). Finally, the effect of the light intensity was investigated irradiating at 365 nm and it turned out to largely effect the kinetic of the reaction (see Supporting Information File 1, Figure S3). In general, the presence of water (Table 1, entries 1 and 2), the addition of oxygen (entries 1, 5, and 6) and light in the UV-A region (entries 8 and 9) turned out to be crucial (see Supporting Information File 1, Table S2).

### Additives

**Figure 1:**
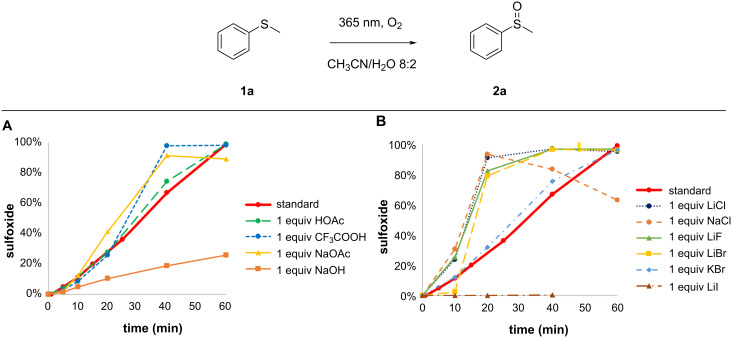
Study of additives. A) Effect of the addition of 1 equiv of various acids and bases to the standard solution. B) Effect of the addition of 1 equiv of various salts to the standard solution.

With the ultimate goal in mind to develop a safe and scalable protocol in continuous flow a study was conducted to explore possible additives that can further enhance the reaction rate (Figure 1) thus increasing the overall productivity. Also, analyzing the effect of the additives on the kinetics might give further clues for the extensive elucidation of the reaction mechanism. For a maximal industrial relevance, the focus was placed on additives that are non-toxic, easy to separate, inexpensive, and readily available. Three classes of compounds have been exploited: acid and bases, salts, and aromatic additives.

#### Acid and bases

As can be seen from Figure 1A, in the presence of strong acids, an increase in reaction rate was observed. For example, when adding 1 equiv trifluoroacetic acid, a full conversion was achieved in 40 minutes instead of 60 minutes. Contrarily, the addition of a strong base substantially slowed down the reaction rate, e.g., the addition of 1 equiv NaOH resulted in only 20% conversion after 60 minutes.

#### Salts

Both LiCl and NaCl induced a strong rate acceleration, resulting in (near) full conversion within only 20 minutes (Figure 1B). With LiCl the selectivity was maintained while the presence of NaCl leads to a decrease in selectivity due to sulfone formation or degradation reactions over longer reaction times. On the other hand, with LiI the reaction was completely quenched. Interestingly, KBr had no effect on the rate and LiBr and LiF also increased the rate, but less than LiCl or NaCl. In general, the addition of salts induced an even stronger effect on the rate than acids and bases and both the anion and cation appear to influence the reaction kinetics.

A deliberate choice of salt can either significantly improve the kinetics or quench the reaction. The latter might be exploited e.g., in late-stage functionalization strategies in order to suppress the photochemical reaction while carrying out another light-induced transformation.

**Aromatic additives** (Table 2): In parallel we observed the significant influence on the reaction rate when the substrate contained an aromatic moiety (e.g., the thioanisole oxidation is over 10 times faster than the oxidation of tetrahydrothiophene). Therefore, we performed a control experiment in the presence of an additive with an aromatic moiety to determine its effect on the reactivity of a non-aromatic substrate. Surprisingly, and to the best of our knowledge, never reported before in literature, the addition of 1 equiv toluene led to 5-fold increase in reaction rate (Table 2, entries 1 and 2). The further study of the impact of electron density on the aromatic ring showed that electron-rich aromatics (such as anisole (Table 2, entry 3)) turned out to be more effective than electron-poor aromatics (e.g., trifluorotoluene (Table 2, entry 4)). Based on these results, the reaction was performed in an anisole/water mixture in order to maximize the effect. This enabled to dramatically increase the reaction rate for the slow reacting tetrahydrothiophene from 7% conversion in 1 hour (no additives) towards full conversion in only 20 minutes.

**Table 2:** Effect of the addition of 1 equivalent of aromatic molecules to the standard solution using tetrahydrothiophene as the substrate.

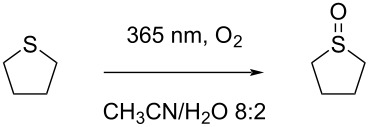				

Entry	Additive	Time	Conversion^a^ (%)	Selectivity^a^ (%)

1	none	60	7	>99
2	toluene	60/120	37/>99	>99/>99
3	anisole	60/120	29/91	>99/>99
4	trifluorotoluene	60/120	21/64	>99/>99
5	anisole as solvent	20	>99	

^a^Based on GC-FID.

### Scope

**Scheme 3:**
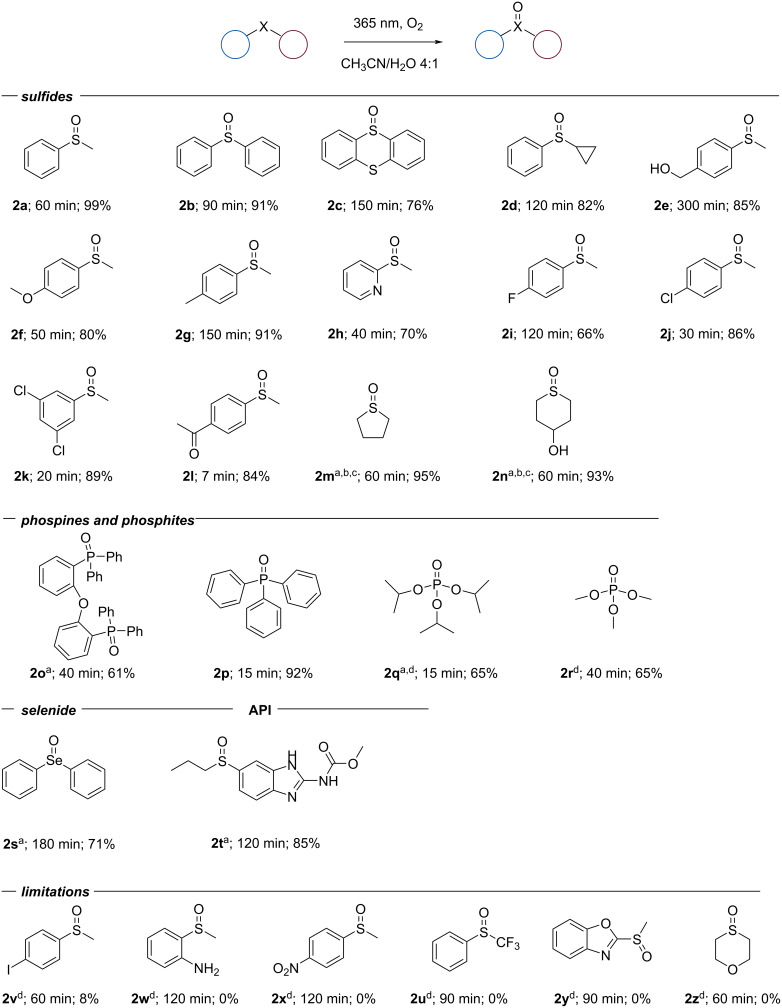
Substrate scope with reaction times and isolated yields. 1 mmol (1 equiv) substrate was reacted in a 5 mL solution of CH_3_CN/H_2_O 8:2 (v:v). The reaction mixture was irradiated with a 365 nm 96 W lamp at a distance of 5 cm from the reactor. Oxygen was bubbled through the solution. ^a^10 mL solution of CH_3_CN/H_2_O 8:2 (v:v). ^b^2 equiv toluene as an additive. ^c^1 equiv LiCl as an additive. ^d^Product not isolated, GC-FID conversion.

Based on the obtained knowledge to optimize the reaction conditions, a variety of sulfides were tested. As shown in Scheme 3, the anticipated products were obtained in good to excellent yields with a few exceptions (**2v**–**z**). In general, heteroatoms at the benzylic position (**2a**–**l**) were easily and readily oxidized selectively and with good functional group tolerance. A slightly slower conversion was observed for more sterically hindered sulfides (**2b**–**d**). For the substrates where kinetics was slower (e.g., sulfides **2m** and **2n** which are lacking an aromatic moiety), there is the option to use an additive (vide supra) to accelerate the reaction.

For products **2f**, **2h**, and **2l** the reaction could be stopped to selectively obtain the sulfoxide, but over prolonged reaction times some side products were observed. As expected, the protocol was not applicable to substrates containing oxygen- or radical-sensitive functionalities (i.e., an amino (**2w**) or nitro group (**2x**)). On the other hand, oxidizable groups, such as alcohols (**2e**), and halogens, such as such as chloro and fluoro on the aromatic ring (**2i** ,**2j**, **2k**), were well tolerated. However, the presence of a iodo group (**2v**) significantly slowed down the reaction. Gratifyingly, we observed excellent selectivity for substrates that contained an additional sulfur atom in the structure. Thus, thianthrene selectively generated product **2c**, which is an important precursor for the synthesis of thianthrene salts [37].

To our delight, the reaction conditions were also applicable to substrates containing other heteroatoms (i.e., P and Se). This allowed us to broaden the scope of the protocol to phosphinoxides (**2o**, **2p**) from phosphines, organophosphates (**2q**, **2r**) from organophosphites, and selenoxide **2s** from selenides.

Finally, to prove the applicability of the reaction conditions in a late-stage functionalization of APIs, the method was carried out on albendazole, and albendazole oxide (**2t**) was obtained with very good yield.

### Flow

The photochemical protocol was then transferred to a flow setup in order to obtain a scalable and thus industrially appealing production method. The oxygenation of triphenylphosphine was used as a model reaction, since the batch results showed fast kinetics (15 minutes). Since triphenylphosphine is insoluble in the reaction mixture we opted to use an oscillatory flow reactor (OFR), specifically the HANU flow reactor (i.e., HANU 2X 5 flow reactor) from Creaflow, as this system can easily handle demanding slurry processes under continuous-flow conditions.

**Scheme 4:**
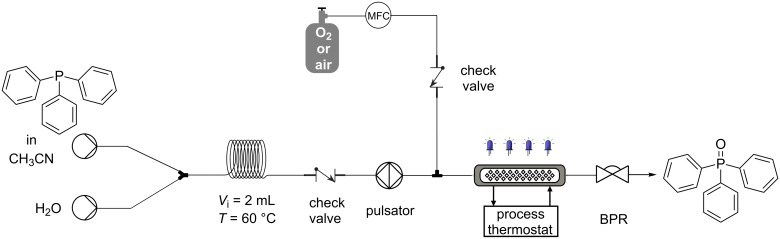
Setup used in the flow experiment for the triphenylphosphine oxidation.

The reaction was carried out using an adapted setup as illustrated in Scheme 4 as triphenylphosphine is very sticky and tends to clog easily in the feeding tubes. This problem was addressed by first solubilizing the substrate in acetonitrile and then mixing the stream (in a 4:1 ratio) with water in a heated mixing loop. This procedure allowed us to pump the substrate in a homogeneous solution into the HANU flow reactor, where it can become a slurry precipitate again, without facing any clogging issues thanks to solid handling capabilities of the flow reactor (Scheme 4). The flow rate and temperature were screened (see Supporting Information File 1, Tables S4 and S5) and it was shown that by transferring the reaction to the described flow conditions, the reaction time could be reduced from 15 minutes in batch to 1 min residence time in flow. To minimize process cost, improve the safety profile, and create a more convenient protocol, the reaction was also carried out using air instead of O_2_. As expected, the residence time increased slightly from 1 minute (oxygen) to 3.5 minutes (air). In order to determine the robustness and overall safety of the flow process, a two-hours run was performed without facing any operational problem.

### Proposed alternative reaction pathway

**Scheme 5:**
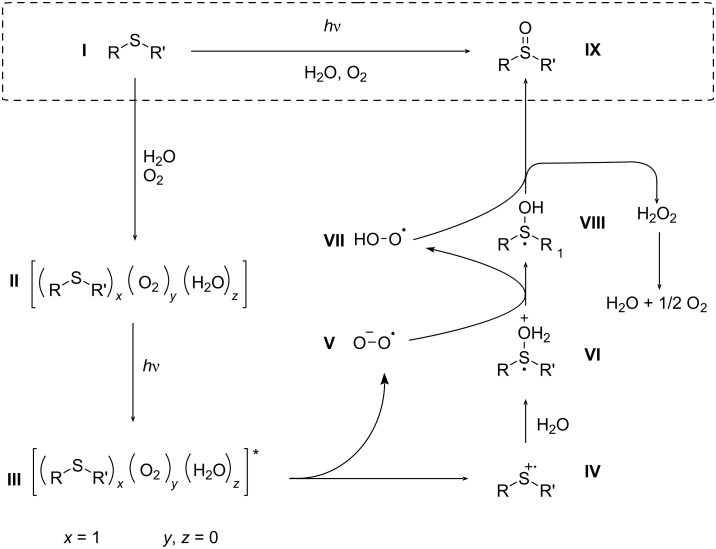
Proposed extra alternative pathway.

In 2017, Bonesi et al. described in detail the oxidation mechanism of sulfides via direct irradiation at 310 nm [27]. They concluded that both a single-electron transfer and a singlet oxygen path can occur depending on the nature of the compound. Intriguingly, the current method applies 365 nm irradiation at which thioanisole does not absorb (Supporting Information File 1, Figure S1). Also, the presence of water has a significant impact on both the kinetics and selectivity. Therefore, we present an extra alternative pathway (Scheme 5), similar to what has been shown in an electrochemical setting for the oxidation of sulfides or selenides [38–41]. Herein, the oxygen in the end product originates from water.

In this tentative mechanism, the sulfide **I** forms with water and oxygen a photoactive complex **II** which is excited at 365 nm towards **III**. Via single-electron transfer both a radical cation **IV** and the superoxide **V** are generated. Subsequently, the sulfide radical cation **IV** undergoes a nucleophilic attack by water. The superoxide first abstracts a proton to form the perhydroxyl radical **VII** followed by hydrogen atom abstraction from intermediate **VIII** to yield sulfoxide **IX**. The generated hydrogen peroxide decomposes into water and oxygen. The novel proposed pathway can either be dominant or negligible depending on the concentration of water.

## Conclusion

A catalyst-free methodology for the selective oxygenation of heteroatoms (S, P, Se) has been developed using only air, water, and light. The protocol allows high conversion and excellent selectivity for a wide scope of substrates, with relative short reaction times. Additionally, benign additives can further enhance the reaction rate. Finally, the protocol was transferred to a continuous-flow setup, making the method scalable and drastically more safe, and thus appealing for implementation in commercial production processes.

## Supporting Information

File 1 General procedures, product characterization, and copies of ^1^H NMR and ^13^C NMR spectra of compounds.
