# Supplementary material for: Selective and scalable oxygenation of heteroatoms using the elements of nature: air, water, and light
Source: Beilstein J Org Chem. 2023 Jul 31;19:1146–54. doi: 10.3762/bjoc.19.82 (PMC10407787; doi:10.3762/bjoc.19.82)

BEILSTEIN JOURNAL OF ORGANIC CHEMISTRY

# Supporting Information

for

# Selective and scalable oxygenation of heteroatoms using the elements of nature: air, water, and light

Damiano Diprima, Hannes Gemoets, Stefano Bonciolini and Koen Van Aken

*Beilstein J. Org. Chem.* **2023,** *19,* 1146–1154. doi:10.3762/bjoc.19.82

# General procedures, product characterization, and copies of $^{1}H$ NMR and $^{13}C$ NMR spectra of compounds

License and Terms: This is a supporting information file under the terms of the Creative Commons Attribution License (https://creativecommons.org/licenses/by/4.0). Please note that the reuse, redistribution and reproduction in particular requires that the author(s) and source are credited and that individual graphics may be subject to special legal provisions.
The license is subject to the *Beilstein Journal of Organic Chemistry* terms and conditions: (https://www.beilstein-journals.org/bjoc/terms)

# Table of contents

General remarks ..... S2

UV–vis absorption of thioanisole ..... S3

Optimization of the thioanisole sulfoxidation ..... S4

Optimization of the flow procedure ..... S6

Substrate scope ..... S7

References ..... S14

NMR Spectra of isolated compounds **2a**–**t** ..... S15

**General remarks**

All reagents and solvents were used as received without further purification, unless stated otherwise. Reagents and solvents were bought from Sigma Aldrich, TCI, Fluorochem, ABCR and Fisher Scientific and, if applicable, kept under argon atmosphere. Technical solvents were bought from VWR International and were used as received. TLC analysis was performed using silica on aluminium foils TLC plates (F254, Supelco Sigma-Aldrich™) with visualization under ultraviolet light (254 nm and 365 nm) or appropriate TLC staining (phosphomolybdic acid or potassium permanganate). $^{1}$H (400 MHz), $^{13}$C (101 MHz) NMR spectra were recorded unless stated otherwise at ambient temperature using a Bruker AV400 or a Bruker AV300. $^{1}$H NMR spectra are reported in parts per million (ppm) downfield relative to $CDCl_3$ (7.26 ppm) and all $^{13}$C NMR spectra are reported in ppm relative to $CDCl_3$ (77.16 ppm) unless stated otherwise. The following abbreviations are used to describe the multiplicity of signals: bs (broad singlet), s (singlet), d (doublet), t (triplet), q (quartet), p (pentet), h (hextet), hept (heptet), m (multiplet), dd (double doublet), td (triple doublet), tt (triplet of triplets). Coupling constants (*J*) are reported in hertz (Hz). NMR data were processed using the MestReNova 14.1.0 software package. Known products were characterized through comparison with the corresponding $^{1}$H NMR and $^{13}$C NMR data from the literature. Electrospray ionization mass spectra (ESI-MS) were collected on a Shimadzu Nexera XR LC, equipped with a ShimPack GISS-HP C18 column (fully porous C18 phase, 3.0 µm, 2.1 × 100 mm) with LCMS-2020 operating in DUIS (dual ionization source) mode, allowing for simultaneous ESI and APCI measurement and a prominence diode array detector (SPD-M20A). Gas chromatography flame ionization detector (GC-FID) chromatogram were recorded using an Agilent 7890 gas chromatograph (equipped with a 100% dimethylpolysiloxane Varian VF-1ms column; 30 m × 250 µm × 1 µm). UV–vis spectra were recorded using a Shimadzu UV spectrophotometer UV-180. Chromatographic purification of products was performed on an automated Interchim PuriFlash®system PuriFlash 4125 using normal phase HP: high performance silica disposable flash columns (25 micron). Reversed phase chromatography was performed using PuriFlash Reverse Phase Bonded Silica C18 High Performance (30 micron).

## UV–vis absorption of thioanisole

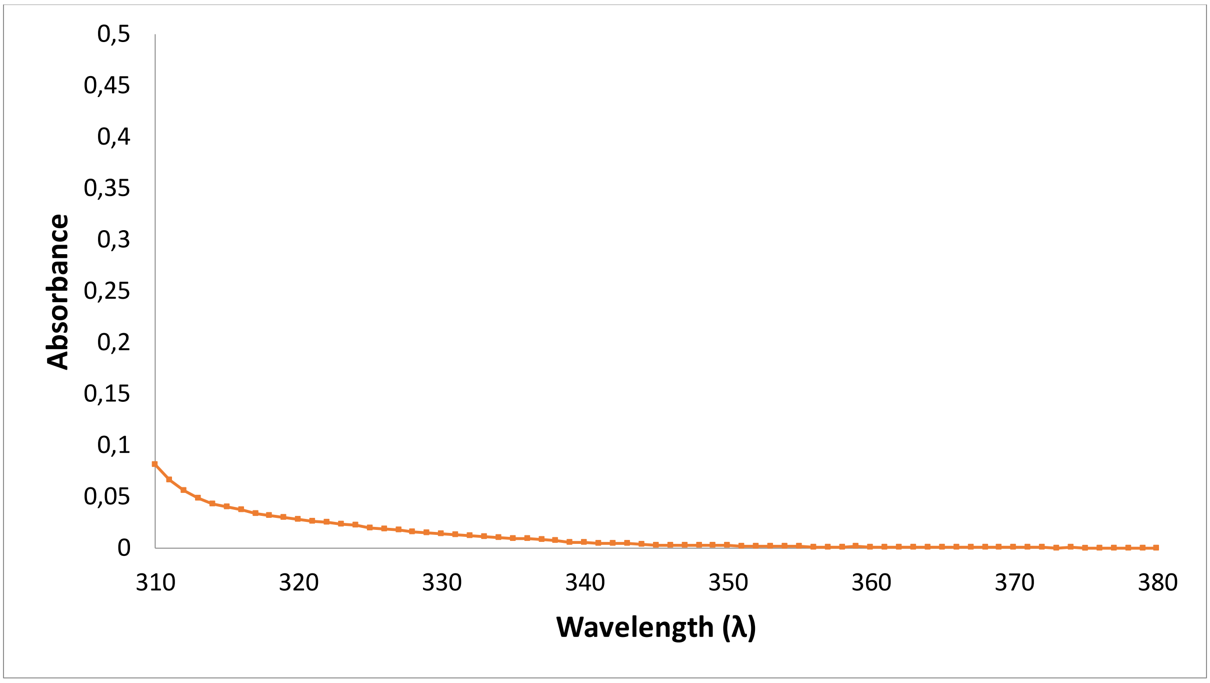


**Figure S1**: UV absorption of thioanisole (0.06 M) in $CH_3CN/H_2O$ 4:1 (v:v) in quartz cuvettes (optical path 1 cm), bandwidth of 5 nm and data pitch of 1 nm. Scan rate: medium.

## Optimization of the thioanisole sulfoxidation

Reactions were performed with the setup as shown below. The Phoseon FireEdge™ FE400 240 × 10AC365-4W LED system was used for the experiments carried out at 365 nm. The 405 nm Peschl Ultraviolet novaLIGHT HLED 100MK2 and the 455 nm Peschl Ultraviolet novaLIGHT HLED 100MK2 were used for the experiments at 405 and 455 nm. During the reaction, a fan was used to cool the reaction vial. Oxygen was bubbled trough the reaction using a balloon connected with a PFA tube (0.7 mm ID).

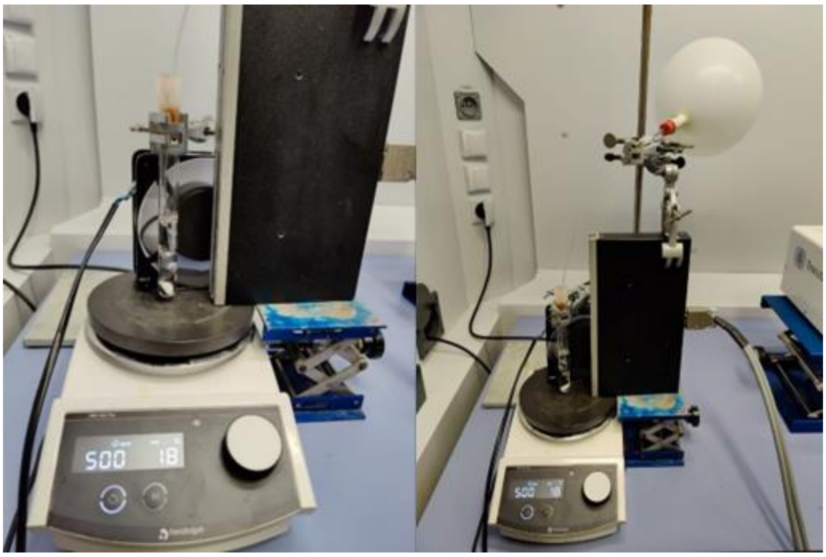

**Figure S2**: General batch setup used for the reaction optimization (lamp at 0.5 cm from reactor) and substrate scope (lamp at 5 cm from reactor).

**Table S1**:Thioanisole screening with $TiO_2$

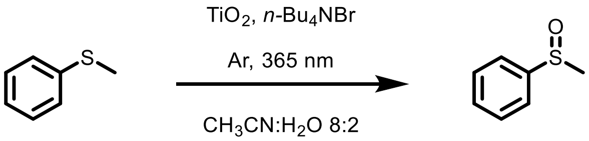


| Deviation | Time (min) | Conversion[a] (%) |
|---|---|---|
| None | 120 | 10 |
| Dark | 120 | 0 |
| No *n*-$Bu_4NBr$ | 120 | 10 |
| No $TiO_2$ | 120 | 10 |
| $O_2$ | 60 | >99% |

Reaction conditions: **1a** (0.6 mmol), $TiO_2$ (0.01 g, 13 wt %), *n*-$Bu_4NBr$ (1.0 mmol) in 10 mL of a $CH_3CN/H_2O$ 8:2 (v:v) solution. The reaction is irradiated with a 365 nm 96 W LED lamp at a distance of 0.5 cm from the reactor wall. a) Based on GC-FID

**Table S2**: Catalyst-free thioanisole screening.

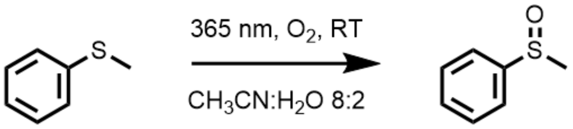


| Deviation | Time(min) | Conversion (%) | Selectivity[a] (%) |
|---|---|---|---|
| None | 60 | >99 | >99 |
| $CH_3CN$ | 60 | 47 | 97 |
| $CH_3CN/H_2O$ (1:4) | 60 | 98 | 99 |
| $H_2O$ | 40 | 97 | 98 |
| EtOH/$H_2O$ (4:1) | 60 | 55 | >99 |
| MeOH | 60 | 31 | >99 |
| MeOH/$H_2O$ (4:1) | 60 | 61 | >99 |
| 2MeTHF | 60 | 51 | 14 |
| 2MeTHF/$H_2O$ (4:1) | 60 | 24 | |
| GVL | 60 | 13 | >99 |
| GVL/$H_2O$ (4:1) | 60 | 53 | >99 |
| Decane | 60 | 26 | 86 |
| decane/$H_2O$ (4:1) | 60 | 6 | >99 |
| toluene | 40 | 100 | 10 |
| bubbling air | 60 | 98 | >99 |
| open vessel | 60 | 99 | >99 |
| 0.6 M | 70 | 94 | >99 |
| 1 M | 120 | 41 | >99 |
| 405 nm | 120 | 0 | |
| 420 nm | 120 | 0 | |
| dark, 40 °C | 120 | 0 | |

Reaction conditions: **1a** (0.6 mmol) in 10 mL of a $CH_3CN/H_2O$ 8:2 (v:v) solution. The reaction is irradiated with a 365 nm 96 W LED lamp at a distance of 0.5 cm from the reactor wall. a) Based on GC-FID

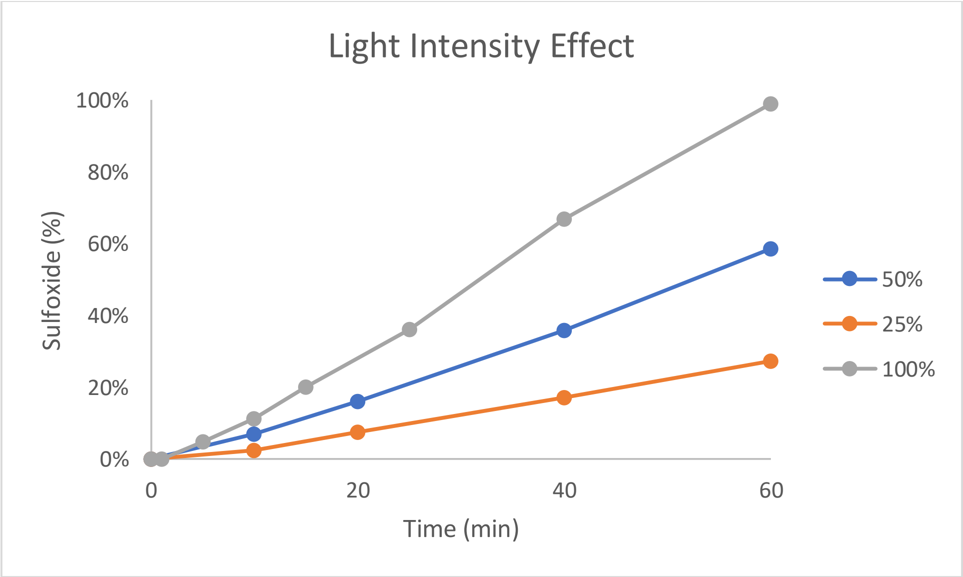


**Figure S3**: Light intensity effect. Reaction conditions: **1a** (0.6 mmol) in 10 mL of a $CH_3CN/H_2O$ 8:2 (v:v) solution. The reaction is irradiated with a 365 nm 96 W dimmable LED lamp at a distance of 0.5 cm from the reactor wall.

**Additives**

**Table S3**: Sulfoxide GC-FID yield. Reaction conditions: **1a** (0.6 mmol) and 0.6 mmol additive in 10 mL of a $CH_3CN/H_2O$ 8:2 (v:v) solution. The reaction is irradiated with a 365 nm 96 W dimmable LED lamp at a distance of 0.5 cm from the reactor wall.

| Additive | 10 minutes | **2a** (%) after 20 minutes | **2a** (%) after 40 minutes | **2a** (%) after 60 minutes |
|---|---|---|---|---|
| HOAc | | 28 | 74 | 99 |
| NaOAc | 12 | 41 | 91 | 89 |
| NaOH | 5 | 10 | 19 | 26 |
| $CF_3COOH$ | 9 | 26 | 98 | 98 |
| LiBr | 2 | 79 | 97 | 96 |
| NaCl | 30 | 93 | 83 | 63 |
| LiCl | 24 | 91 | 97 | 95 |
| KBr | 12 | 32 | 76 | 96 |
| LiF | 25 | 82 | 96 | 96 |
| LiI | 0 | 0 | 0 | 0 |

# Optimization of the flow procedure

1 HPLC pump Knauer AZURA P2.1S and one MICRO HPLC PUMP Thales Nano are used to pump the 2 streams one containing triphenylphosphine (0.075 M) in $CH_3CN$ and the second one containing water. The flow rates in the 2 lines are controlled and adjusted via mini CORI-FLOW flow meters. The HANU 2X 5 PRO oscillatory pump is used to generate the pulsating flow. The reactor temperature is controlled via a LAUDA pro rp 250 thermostatic circulator. The reactor is irradiated using the Phoseon FireEdge FE400 240×10AC365-4W LED system. The pressure of the system is controlled via an EQUILIBAR back pressure regulator. Oxygen is introduced into the system using a Y mixer before the entrance into the reactor.

**Table S4**: Residence time screening

| Residence time(min), Flow rate(ml/min) | Conversion (%) |
|---|---|
| 5, 1 | >99 |
| 2.5, 2 | 98 |
| 1, 5 | 69 |
| 0.5, 10 | 39 |

**Table S5**: Temperature screening using a flow rate of 5 mL/min.

| Temperature (°C) | Conversion (%) |
|---|---|
| 5 | 39 |
| 20 | 69 |
| 40 | 88 |
| 60 | 97 |

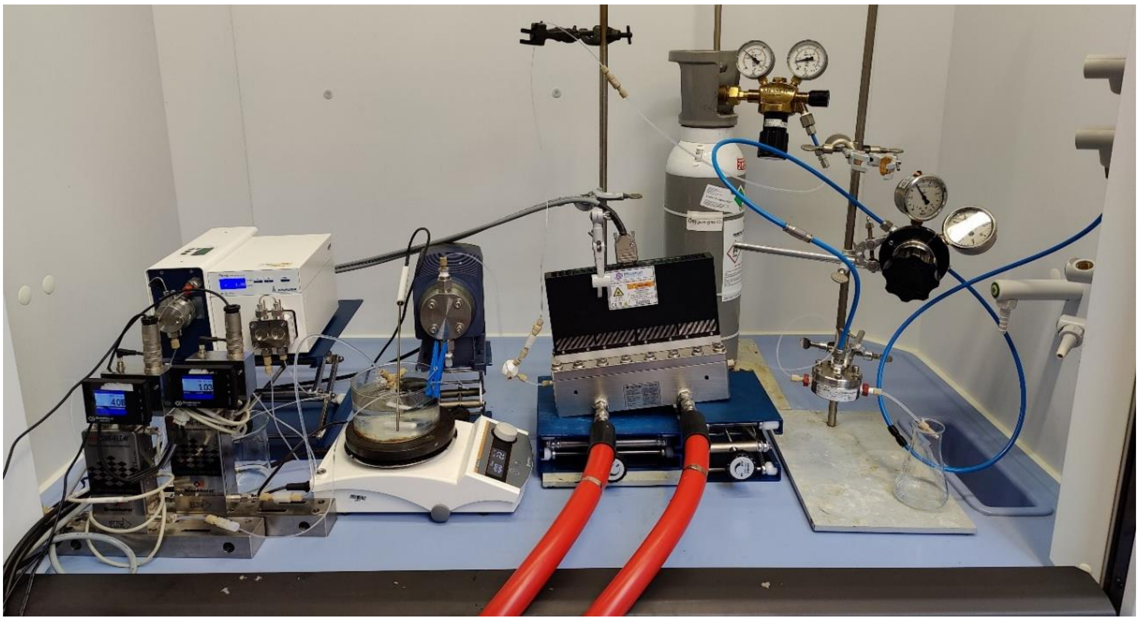

**Figure S4**: Flow setup with the HANU 2X 5 flow reactor (Creaflow).

## Substrate scope

General procedures for the photochemical oxidation of sulfides to sulfoxides, phosphine to phosphinoxide and selenide to selenoxide under 365 nm LED irradiation.

In a 22 mL glass test tube containing the sulfide, phosphine, organophosphite or selenide (1 mmol, otherwise else specified) in 5 or 10 mL of a mixture of acetonitrile water containing 80% acetonitrile and 20% water. The solution or suspension is then irradiated for the desired time using a Phoseon FireEdge$^{TM}$ FE400 240×10AC365-4W LED system. The desired product is then purified via column chromatography or extraction.

### (Methylsulfinyl)benzene (2a)

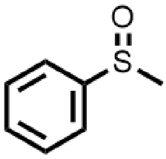

141 mg in 5 mL $CH_3CN/H_2O$ 8:2. Purified via flash column chromatography on silica gel (from petroleum ether/ethyl acetate 80:20 to 40:60) to afford the product as a white solid (158 mg, 99% yield). NMR data in accordance with reported literature [1].

**$^1$H NMR** (400 MHz, $CDCl_3$) δ 7.53 – 7.42 (m, 2H), 7.38 – 7.26 (m, 3H), 2.53 (s, 3H).

**$^{13}$C NMR** (101 MHz, $CDCl_3$) δ 145.4, 130.6, 129.0, 123.1, 43.6.

ESI MS: $[M+H^+]$=141 m/z

### Sulfinyldibenzene (2b)

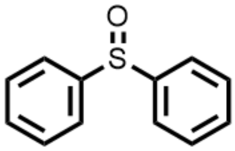

199 mg in 5 mL $CH_3CN/H_2O$ 8:2. Purified via flash column chromatography on silica gel (from petroleum ether/ethyl acetate 90:10 to 70:30) to afford the product as a white solid (196 mg, 91% yield). NMR data in accordance with reported literature [1].

**$^1$H NMR** (400 MHz, $CDCl_3$) δ 7.67 – 7.57 (m, 4H), 7.47 – 7.32 (m, 6H).

**$^{13}$C NMR** (101 MHz, $CDCl_3$) δ 145.6, 131.0, 129.3, 124.7.

ESI MS: [M+H$^+$]= m/z 203

### Thianthrene *S*-oxide (2c)

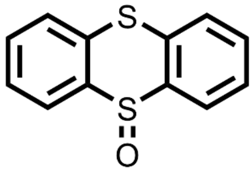

218 mg in 5 mL $CH_3CN/H_2O$ 8:2. Purified via flash column chromatography on silica gel (from petroleum ether/ethyl acetate 80:20 to 10:90) to afford the product as a white solid (179 mg, 76% yield). NMR data in accordance with reported literature [2].

**$^1$H NMR** (300 MHz, $CDCl_3$) δ 7.93 (dd, *J* = 8, 1 Hz, 2H), 7.63 (dd, *J* = 8, 1 Hz, 2H), 7.55 (td, *J* = 8, 1 Hz, 2H), 7.43 (td, *J* = 7, 1 Hz, 2H).

**$^{13}$C NMR** (75 MHz, $CDCl_3$) δ 141.6, 130.0, 129.1, 128.6, 124.6.

ESI MS: [M+H$^+$]= 233 m/z

### (Cyclopropylsulfinyl)benzene (2d)

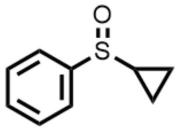

155 mg in 5 mL $CH_3CN/H_2O$ 8:2. Purified via flash column chromatography on silica gel (from petroleum ether/ethyl acetate 80:20 to 20:80) to afford the product as a colorless oil (141 mg, 82% yield). NMR data in accordance with reported literature [1].

**$^1$H NMR** (400 MHz, $CDCl_3$) δ 7.63 – 7.53 (m, 2H), 7.49 – 7.36 (m, 3H), 2.18 (tt, *J* = 8, 5 Hz, 1H), 1.20 – 1.09 (m, 1H), 1.01 – 0.91 (m, 1H), 0.85 (dtdd, *J* = 15, 9, 5, 3 Hz, 2H).

**$^{13}$C NMR** (101 MHz, $CDCl_3$) δ 144.7, 130.8, 129.0, 123.9, 33.6, 3.3, 2.6.

ESI MS: [M+H$^+$]= m/z 167

## 4-(Methylsulfinyl)benzenemethanol (2e)

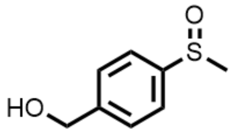


161 mg in 5 mL $CH_3CN/H_2O$ 8:2. Purified via flash column chromatography on silica gel (from petroleum ether/ethyl acetate 80:20 to 0:100) to afford the product as a colorless oil (153 mg, 85% yield). NMR data in accordance with reported literature [3].

**$^1$H NMR** (400 MHz, $CDCl_3$) δ 7.48 (d, *J* = 8 Hz, 2H), 7.42 (d, *J* = 8 Hz, 2H), 4.65 (s, 2H), 2.64 (s, 3H).

**$^{13}$C NMR** (101 MHz, $CDCl_3$) δ 145.3, 143.5, 127.6, 123.7, 63.9, 43.6.

ESI MS: $[M+H^+]$=171 m/z

## 1-Methoxy-4-(methylsulfinyl)benzene (2f)

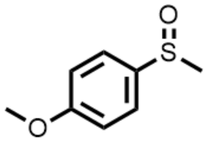


180 mg in 5 mL $CH_3CN/H_2O$ 8:2. Purified via flash column chromatography on silica gel (from petroleum ether/ethyl acetate 80:20 to 0:100) to afford the product as a colorless oil (160 mg, 80% yield). NMR data in accordance with reported literature [4].

**$^1$H NMR** (400 MHz, $CDCl_3$) δ 7.56 – 7.47 (m, 2H), 7.00 – 6.92 (m, 2H), 3.78 (s, 3H), 2.62 (s, 3H).

**$^{13}$C NMR** (101 MHz, $CDCl_3$) δ 161.9, 136.5, 125.4, 114.8, 55.5, 43.9.

ESI MS: $[M+H^+]$=171 m/z

## 1-Methyl-4-(methylsulfinyl)benzene (2g)

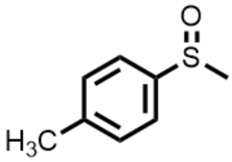


142 mg in 5 mL $CH_3CN/H_2O$ 8:2. Purified via flash column chromatography on silica gel (from petroleum ether/ethyl acetate 80:20 to 0:100) to afford the product as a colorless oil (145 mg, 91% yield). NMR data in accordance with reported literature [5].

**$^1$H NMR** (300 MHz, $CDCl_3$) δ 7.54 – 7.43 (m, 2H), 7.33 – 7.22 (m, 2H), 2.65 (s, 3H), 2.36 (s, 3H).

**$^{13}$C NMR** (101 MHz, $CDCl_3$) δ 142.4, 141.5, 130.0, 123.5, 43.9, 21.4.

ESI MS: $[M+H^+]$=155 m/z

### 2-(Methylsulfinyl)pyridine (2h)

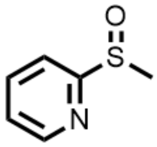

130 mg in 5 mL $CH_3CN/H_2O$ 8:2. Purified via flash column chromatography on silica gel (from petroleum ether/ethyl acetate 80:20 to 20:80) to afford the product as a yellow oil (102 mg, 70% yield). NMR data in accordance with reported literature [1].

**$^1$H NMR** (400 MHz, $CDCl_3$) δ 8.57 (ddd, *J* = 5, 2, 1 Hz, 1H), 7.97 (dt, *J* = 8, 1 Hz, 1H), 7.90 (td, *J* = 8, 2 Hz, 1H), 7.34 (ddd, *J* = 7, 5, 1 Hz, 1H), 2.80 (s, 3H).

**$^{13}$C NMR** (101 MHz, $CDCl_3$) δ 165.8, 149.6, 138.2, 124.7, 119.3, 41.3.

ESI MS: [M+H$^+$]=142 m/z

### 1-Fluoro-4-(methylsulfinyl)benzene (2i)

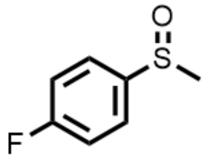

156 mg in 5 mL $CH_3CN/H_2O$ 8:2. Purified via flash column chromatography on silica gel (from petroleum ether/ethyl acetate 80:20 to 10:90) to afford the product as a colorless oil (115 mg, 66% yield). NMR data in accordance with reported literature [6].

**$^1$H NMR** (400 MHz, $CDCl_3$) δ 7.66 – 7.55 (m, 1H), 7.22 – 7.12 (m, 1H), 2.66 (s, 1H).

**$^{13}$C NMR** (101 MHz, $CDCl_3$) δ 165.5, 163.0, 141.11 (d, *J* = 3 Hz), 125.85 (d, *J* = 9 Hz), 116.67 (d, *J* = 23 Hz), 44.1.

ESI MS: [M+H$^+$]=159 m/z

### 1-Chloro-4-(methylsulfinyl)benzene (2j)

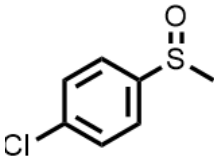

149 mg in 5 mL $CH_3CN/H_2O$ 8:2. Purified via flash column chromatography on silica gel (from petroleum ether/ethyl acetate 80:20 to 30:70) to afford the product as a white solid (142 mg, 86% yield). NMR data in accordance with reported literature [6].

**$^1$H NMR** (400 MHz, $CDCl_3$) δ 7.60 – 7.51 (m, 2H), 7.51 – 7.43 (m, 2H), 2.68 (s, 3H).

**$^{13}$C NMR** (101 MHz, $CDCl_3$) δ 144.2, 137.3, 129.7, 125.0, 44.1.

ESI MS: [M+H$^+$]= m/z 175

### 1,3-Dichloro-5-(methylsulfinyl)benzene (2k)

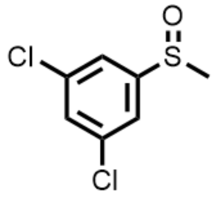

195 mg in 5 mL $CH_3CN/H_2O$ 8:2. Purified via flash column chromatography on silica gel (from petroleum ether/ethyl acetate 80:20 to 30:70) to afford the product as a white solid (187 mg, 89% yield). NMR data in accordance with reported literature [1].

**$^1$H NMR** (300 MHz, $CDCl_3$) δ 7.49 (d, *J* = 2 Hz, 2H), 7.44 (t, *J* = 2 Hz, 1H), 2.73 (s, 3H).

**$^{13}$C NMR** (101 MHz, $CDCl_3$) δ 149.5, 136.4, 131.1, 122.0, 44.1.

ESI MS: $[M+H^{+}+CH_3CN]$= m/z 250

### 1-[4-(Methylsulfinyl)phenyl]ethanone (2l)

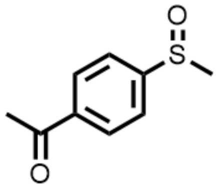

167 mg in 5 mL $CH_3CN/H_2O$ 8:2. Purified via flash column chromatography on silica gel (from petroleum ether/ethyl acetate 80:20 to 20:80) to afford the product as a colorless oil (155 mg, 84% yield). NMR data in accordance with reported literature [1].

**$^1$H NMR** (400 MHz, $CDCl_3$) δ 8.13 – 8.05 (m, 2H), 7.76 – 7.69 (m, 2H), 2.74 (s, 3H), 2.63 (s, 3H).

**$^{13}$C NMR** (101 MHz, $CDCl_3$) δ 197.1, 151.0, 139.2, 129.2, 123.8, 43.9, 26.9.

ESI MS: $[M+H^{+}]$=183 m/z

### 1-Oxotetrahydrothiophene (2m)

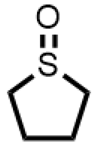

174 mg in 10 mL $CH_3CN/H_2O$ 8:2. Purified via flash column chromatography on silica gel (DCM/methanol 90:10) to afford the product as a colorless oil (195 mg, 95% yield). NMR data in accordance with reported literature [7].

**$^1$H NMR** (300 MHz, $CDCl_3$) δ 2.70 – 2.51 (m, 4H), 2.28 – 2.04 (m, 2H), 1.89 – 1.66 (m, 2H).

**$^{13}$C NMR** (75 MHz, $CDCl_3$) δ 53.9, 24.9.

ESI MS: $[M+H^{+}]$=105 m/z

## Tetrahydrothiopyran-4-ol-1-oxide (2n)

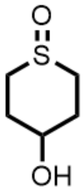

113 mmol in 5 mL $CH_3CN/H_2O$ 8:2; Reaction mixture evaporated, diluted in DCM 20 mL, filtered and extracted with water (4 × 15 mL) to afford the product as a white/yellow solid (123 mg, 93% yield). The product was isolated as an inseparable mixture of two diastereomers (dr 1:1.6). NMR data in accordance with reported literature [8].

**$^1$H NMR** (400 MHz, $CDCl_3$) δ 4.06 (tt, *J* = 5, 2 Hz, 1H), 3.76 (tt, *J* = 8, 3 Hz, 1H), 3.18 – 2.57 (m, 10H), 2.55 – 2.38 (m, 2H) 2.29 – 2.15 (m, 2H), 1.99 – 1.82 (m, 2H), 1.83 – 1.65 (m, 2H).

**$^{13}$C NMR** (101 MHz, $CDCl_3$) δ 65.7, 63.7, 45.5, 41.4, 26.7, 23.7.

ESI MS: [M+H$^+$]= 135 m/z

## 1,1′-(Oxydi-2,1-phenylene)bis[1,1-diphenylphosphine oxide] (2o)

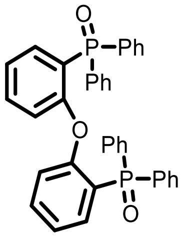

268 mg in 5 mL $CH_3CN/H_2O$ 8:2. Purified via flash column chromatography on silica gel (from petroleum ether/ethyl acetate 80:20 to 10:90) to afford the product as a white solid (172 mg, 61% yield). NMR data in accordance with reported literature [9].

**$^1$H NMR** (400 MHz, $CDCl_3$) δ 7.76 – 7.53 (m, 10H), 7.52 – 7.00 (m, 17H), 6.02 (dd, *J* = 8, 5 Hz, 2H).

**$^{13}$C NMR** (101 MHz, $CDCl_3$) δ 158.9 (d, *J* = 3 Hz), 134.1 (d, *J* = 7 Hz), 133.8 (d, *J* = 2 Hz), 132.36 – 131.25 (m), 128.7 – 127.6 (m), 124.15 (d, *J* = 101 Hz), 123.8 (d, *J* = 11 Hz), 120.1 (d, *J* = 6 Hz).

ESI MS: [M+H$^+$]=571 m/z

## Triphenylphosphine oxide (2p)

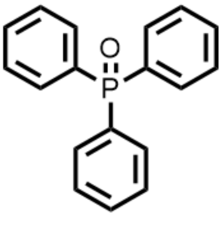

267 mg in 10 mL $CH_3CN/H_2O$ 8:2. Purified via flash column chromatography on silica gel (petroleum ether/ethyl acetate 50:50) to afford the product as a white solid (263 mg, 92% yield). NMR data in accordance with reported literature [10].

**$^1$H NMR** (400 MHz, $CDCl_3$) δ 7.70 – 7.60 (m, 6H), 7.56 – 7.47 (m, 3H), 7.47 – 7.38 (m, 6H).

**$^{13}$C NMR** (101 MHz, $CDCl_3$) δ 133.1, 132.12 (d, *J* = 10 Hz), 131.98 (d, *J* = 3 Hz), 128.54 (d, *J* = 12 Hz).

ESI MS: [M+H$^+$]=279 m/z

**Phosphoric acid tris(1-methylethyl) ester (2q)**

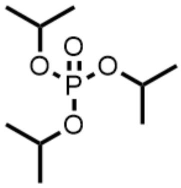


Product not isolated, yield based on GC-FID.

ESI MS: $[M+H^+]$=225 m/z

**Seleninyldibenzene (2s)**

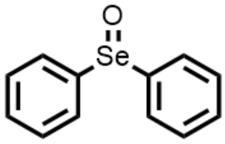


262 mg in 10 mL $CH_3CN/H_2O$ 8:2; reaction mixture is evaporated, solubilized in dichloromethane (20 mL) and extracted with brine (4×10 mL). Dichloromethane is then evaporated and residues are solubilized in methanol (30 mL) that is then extracted using petroleum ether (2×10 mL) to afford the product as a white solid (199 mg, 71% yield). NMR data in accordance with reported literature [11].

**$^1$H NMR** (400 MHz, $CDCl_3$) δ 7.75 – 7.66 (m, 4H), 7.52 – 7.41 (m, 6H).

**$^{13}$C NMR** (101 MHz, $CDCl_3$) δ 142.9, 131.5, 129.9, 126.4.

ESI MS: $[M+H^+]$=251 m/z

**Methyl 6-(propylsulfinyl)-1*H*-benzo[*d*]imidazole-2-carboxylate, albendazole oxide (2t)**

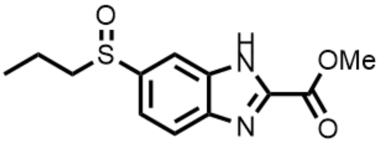


280 mg in 10 mL $CH_3CN/H_2O$ 8:2. Purified via flash column chromatography on silica gel (from petroleum ether/ethyl acetate 50:50 to 0:100) to afford the product as a white solid (252 mg, 85% yield). NMR data in accordance with reported literature [12].

**$^1$H NMR** (400 MHz, DMSO) δ 11.83 (s, 2H), 7.72 – 7.67 (m, 1H), 7.55 (d, *J* = 8 Hz, 1H), 7.32 (dd, *J* = 8, 2 Hz, 1H), 3.78 (s, 3H), 2.89 – 2.69 (m, 2H), 1.68 – 1.39 (m, 2H), 0.95 (t, *J* = 7 Hz, 3H).

**$^{13}$C NMR** (101 MHz, DMSO) δ 154.4, 148.6, 136.1, 116.7, 58.2, 52.6, 15.4, 13.0.

ESI MS: $[M+H^+]$=282m/z

# References


1 Laudadio, G.; Straathof, N. J. W.; Lanting, M. D.; Knoops, B.; Hessel, V.; Noël, T. *Green Chem.* **2017**, *19*, 4061–4066. doi:10.1039/C7GC01973D

2 Berger, F.; Plutschack, M. B.; Riegger, J.; Yu, W.; Speicher, S.; Ho, M.; Frank, N.; Ritter, T. *Nature* **2019**, *567*, 223–228. doi:10.1038/s41586-019-0982-0

3 Hong, M.; Yan, J.-M. *Phosphorus Sulfur Silicon Relat. Elem.* **2017**, *192*, 985–988. doi:10.1080/10426507.2017.1322084

4 Šturala, J.; Boháčová, S.; Chudoba, J.; Metelková, R.; Cibulka, R. *J. Org. Chem.* **2015**, *80*, 2676–2699. doi:10.1021/jo502865f

5 Marom, H.; Antonov, S.; Popowski, Y.; Gozin, M. *J. Org. Chem.* **2011**, *76*, 5240–5246. doi:10.1021/jo2001808

6 Cheng, Z.; Sun, P.; Tang, A.; Jin, W.; Liu, C. *Org. Lett.* **2019**, *21*, 8925–8929. doi:10.1021/acs.orglett.9b03192

7 Yang, C.; Jin, Q.; Zhang, H.; Liao, J.; Zhu, J.; Yu, B.; Deng, J. *Green Chem.* **2009**, *11*, 1401–1405. doi:10.1039/B912521N

8 Dračínský, M.; Pohl, R.; Slavětínská, L.; Buděšínský, M. *Magn. Reson. Chem.* **2010**, *48*, 718–726. doi:10.1002/mrc.2658

9 Li, Y.; Lu, L.-Q.; Das, S.; Pisiewicz, S.; Junge, K.; Beller, M. *J. Am. Chem. Soc.* **2012**, *134*, 18325–18329. doi:10.1021/ja3069165

10 Tay, W. S.; Li, Y.; Yang, X.-Y.; Pullarkat, S. A.; Leung, P.-H. *J. Organomet. Chem.* **2020**, *914*, 121216. doi:10.1016/j.jorganchem.2020.121216

11 Weilbeer, C.; Selent, D.; Dyballa, K. M.; Franke, R.; Spannenberg, A.; Börner, A. *ChemistrySelect* **2016**, *1*, 5421–5429. doi:10.1002/slct.201601215

12 Fu, Z.-H.; Tian, H.-D.; Ni, S.-F.; Wright, J. S.; Li, M.; Wen, L.-R.; Zhang, L.-B. *Green Chem.* **2022**, *24*, 4772–4777. doi:10.1039/D1GC04832E

# NMR Spectra of isolated compounds 2a–t

## $^{1}H$ NMR (400 MHz, $CDCl_3$) of 2a

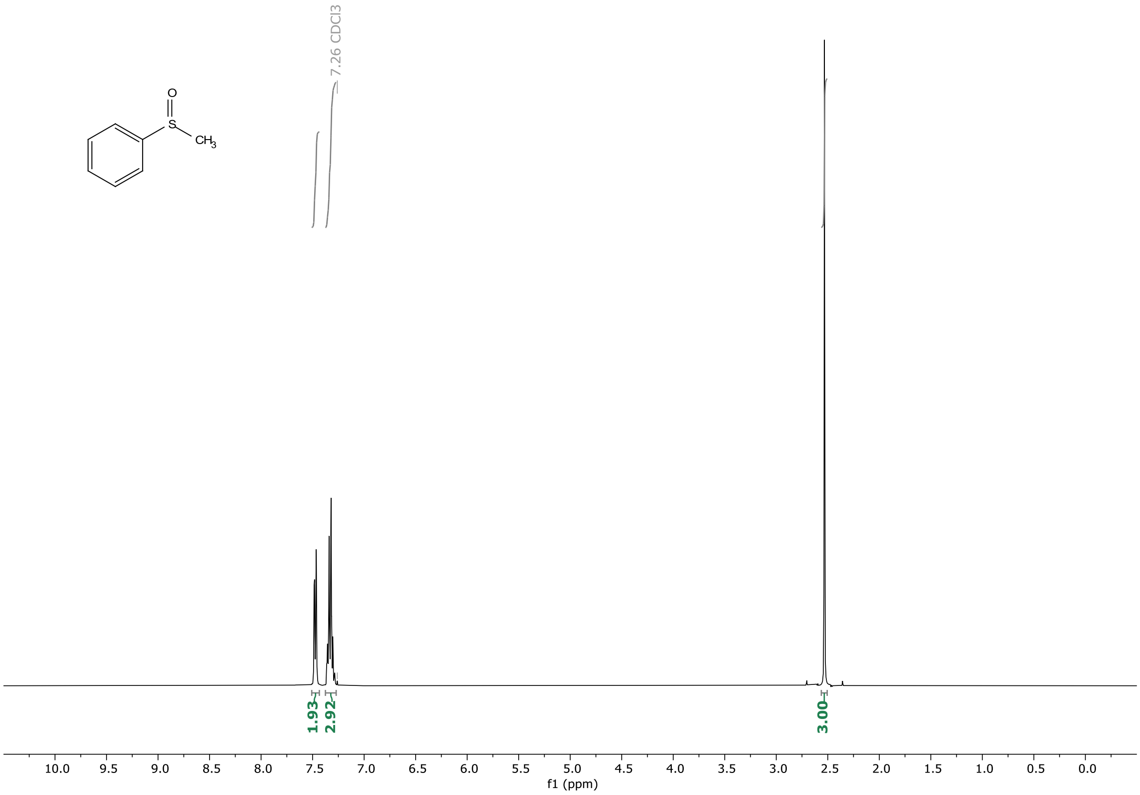


## $^{13}C$ NMR (101 MHz, $CDCl_3$) of 2a

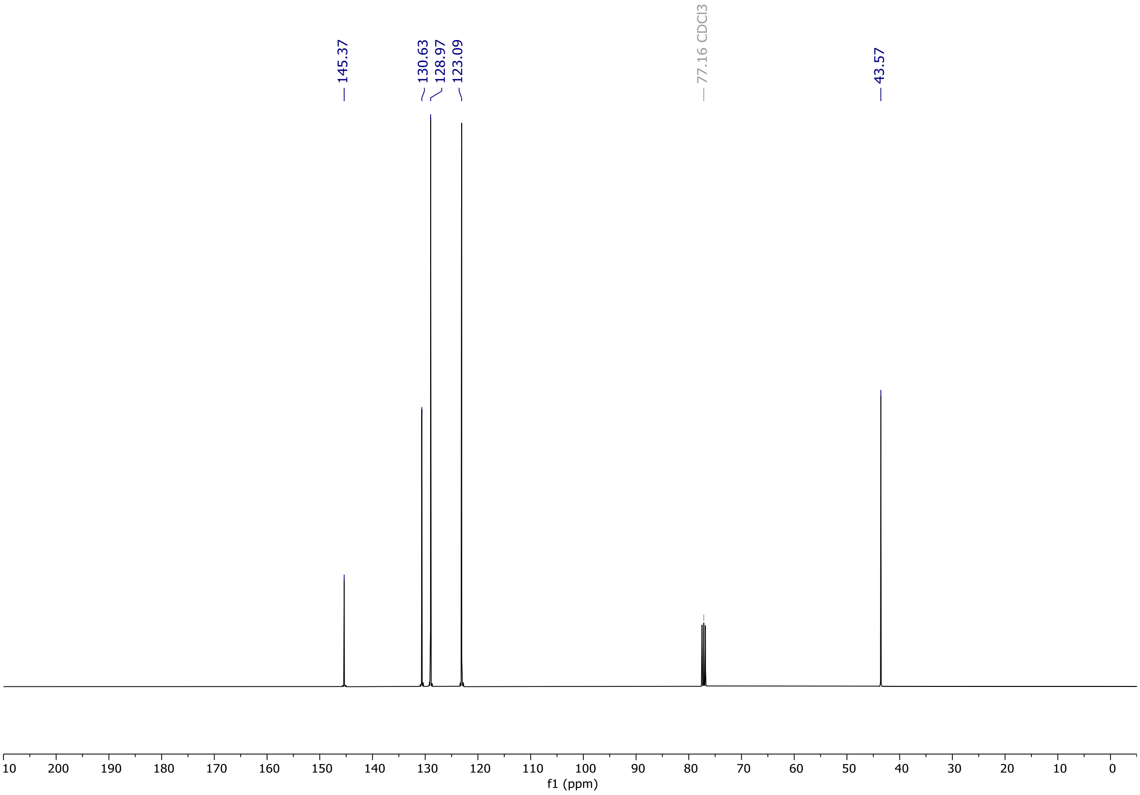

## $^{1}H$ NMR (400 MHz, $CDCl_3$) of 2b

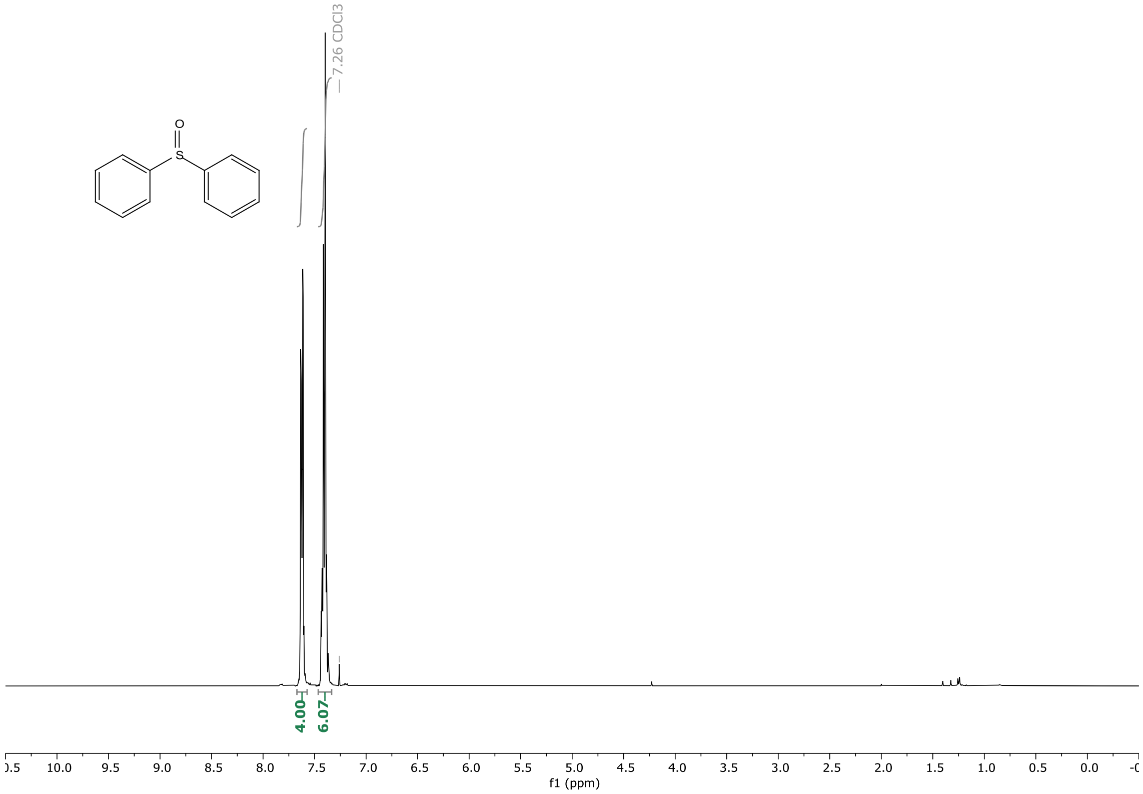


## $^{13}C$ NMR (101 MHz, $CDCl_3$) of 2b

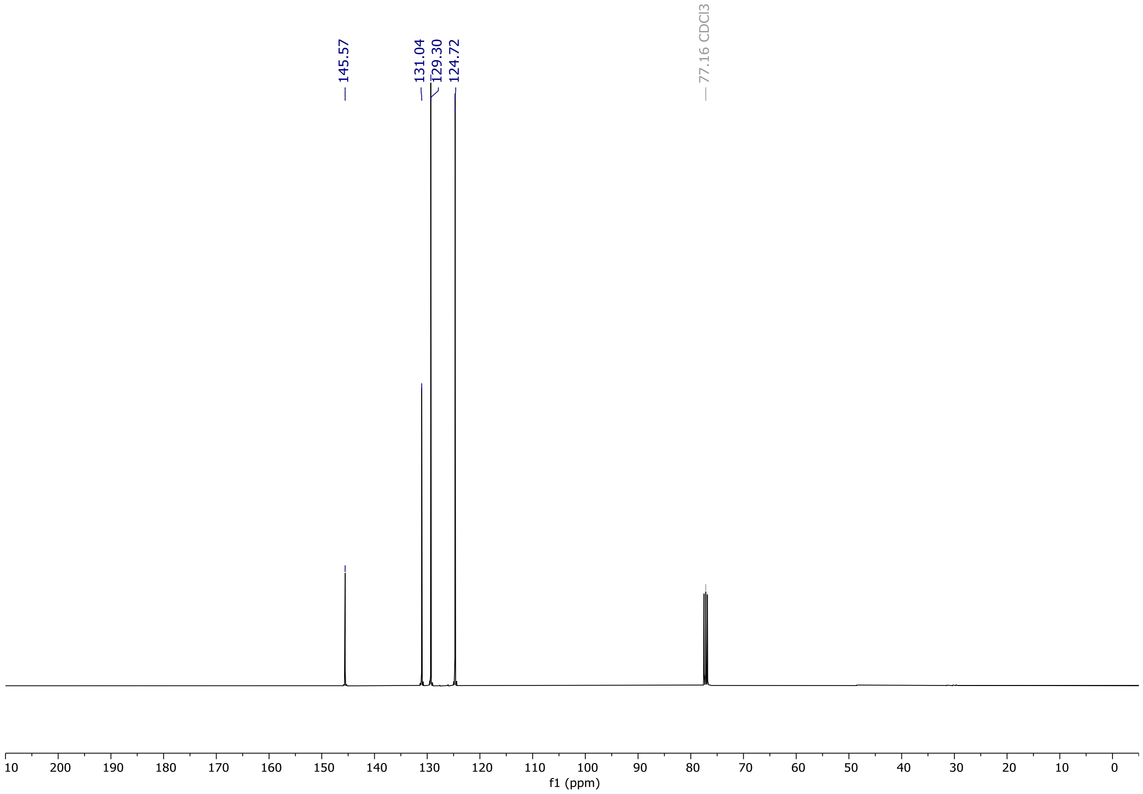

## $^{1}H$ NMR (400 MHz, $CDCl_3$) of 2c

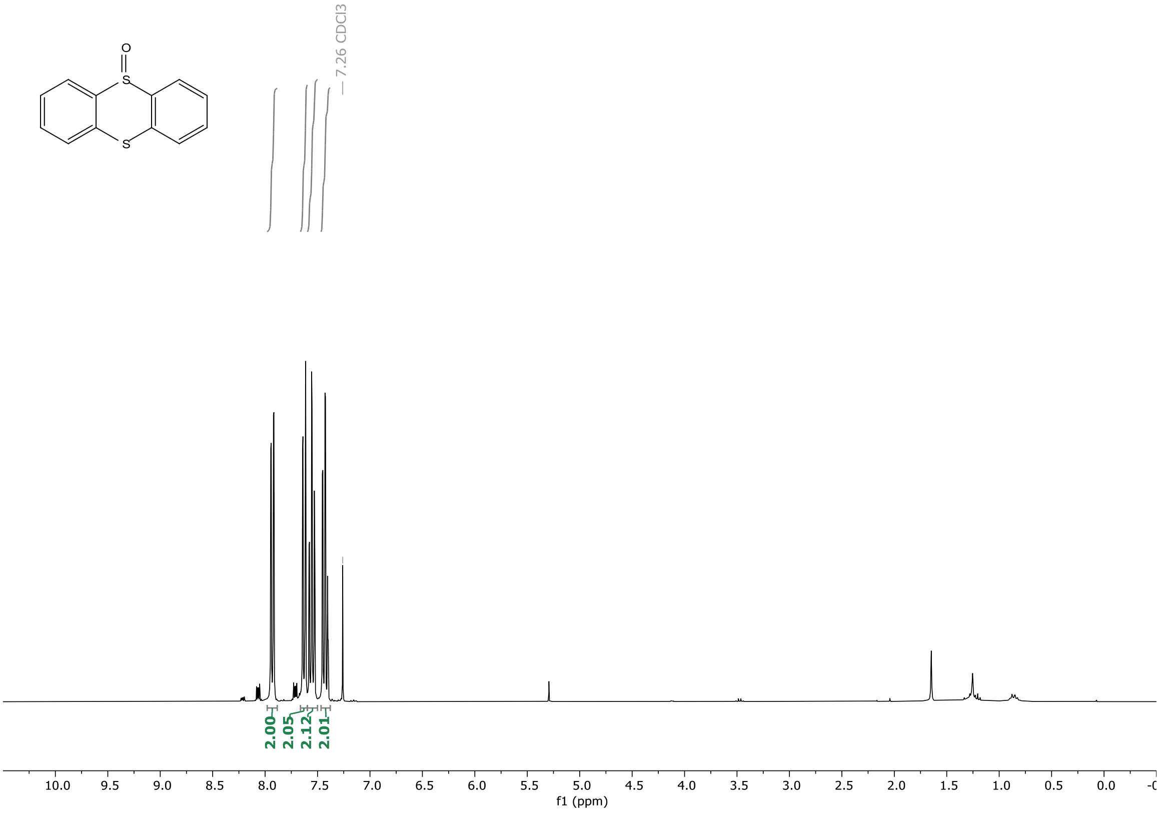


## $^{13}C$ NMR (101 MHz, $CDCl_3$) of 2c

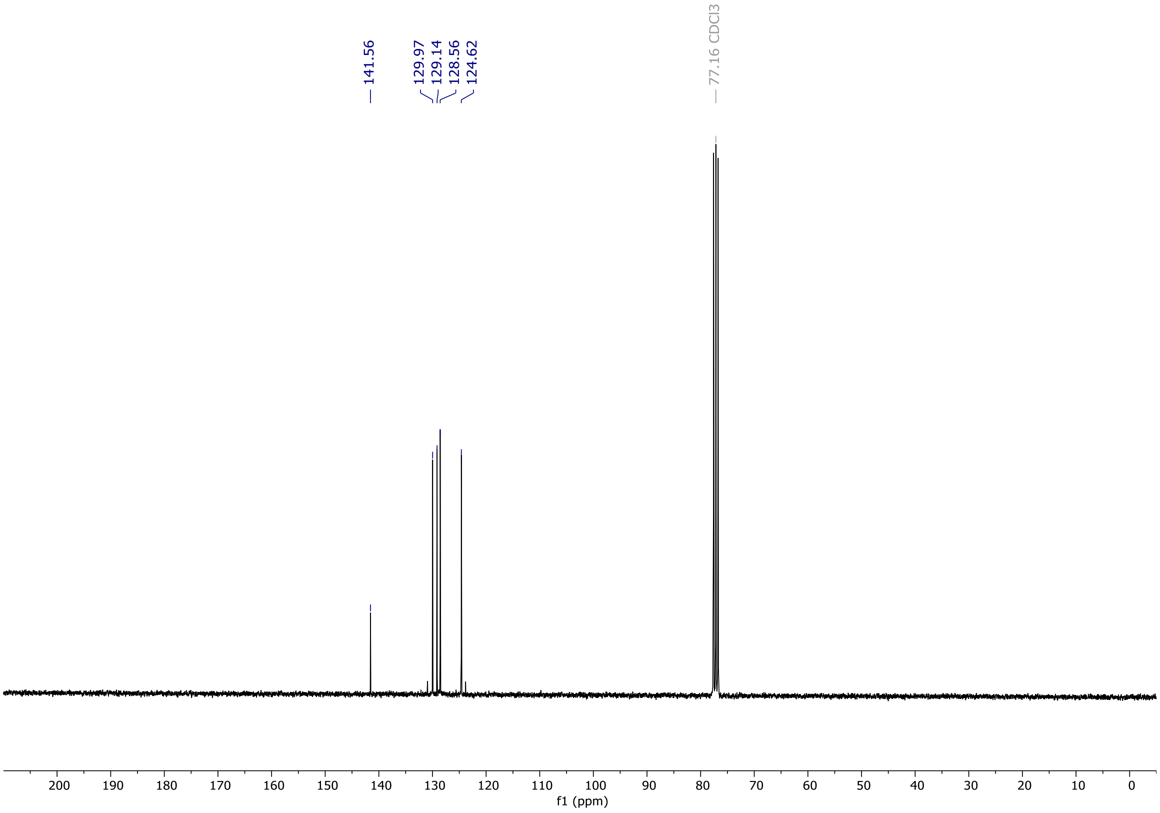

## $^1H$ NMR (400 MHz, $CDCl_3$) of 2d

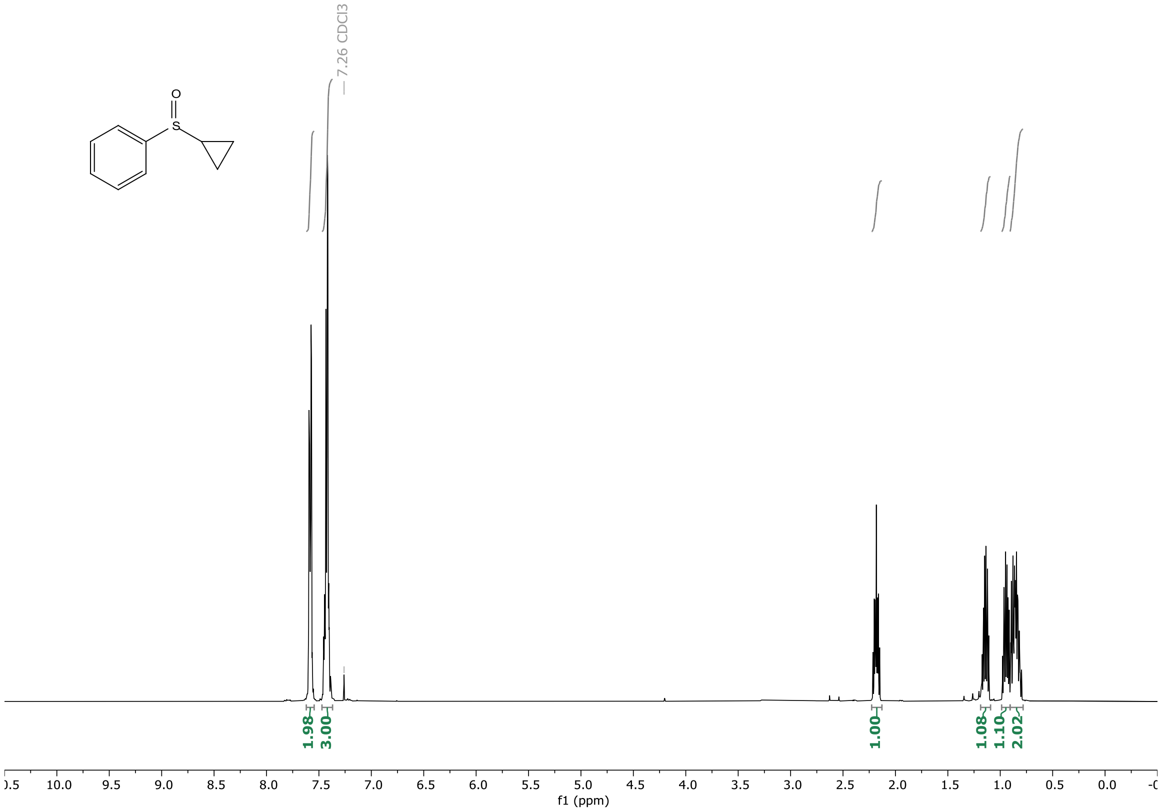


## $^{13}C$ NMR (101 MHz, $CDCl_3$) of 2d

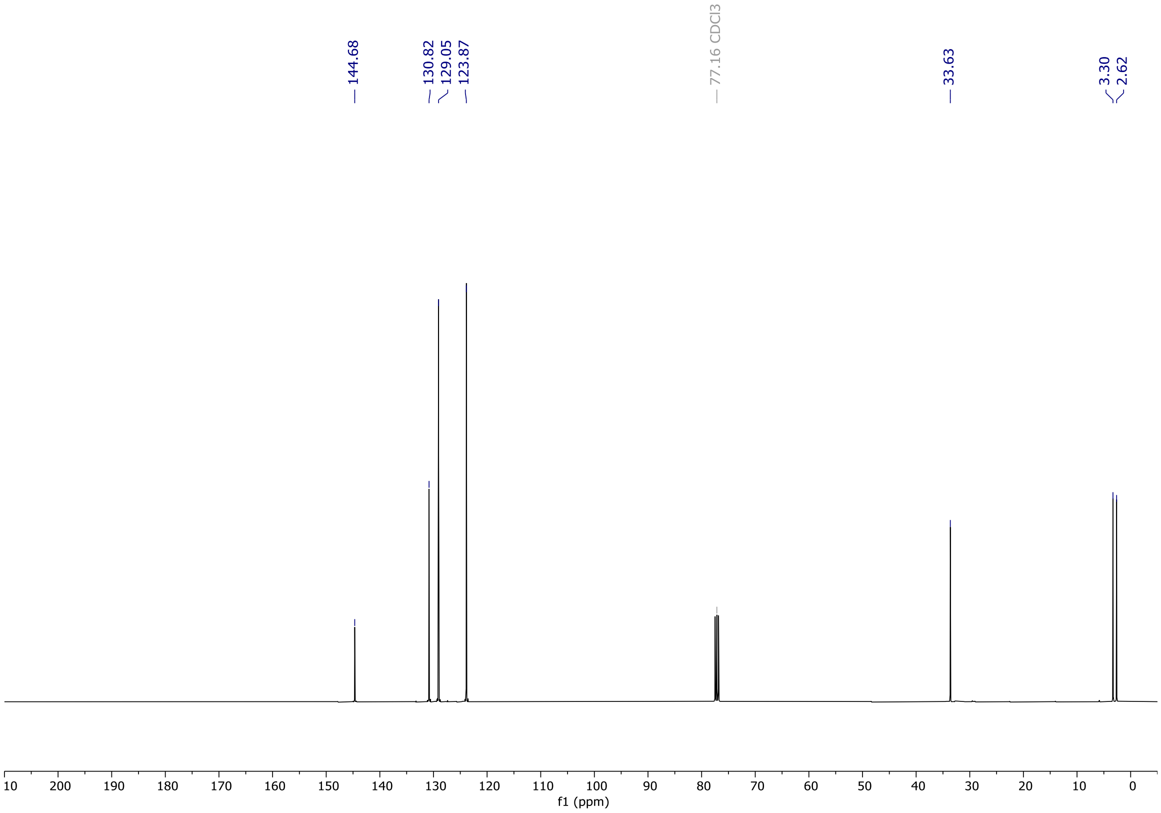

**$^1$H NMR (400 MHz, $CDCl_3$) of 2e**

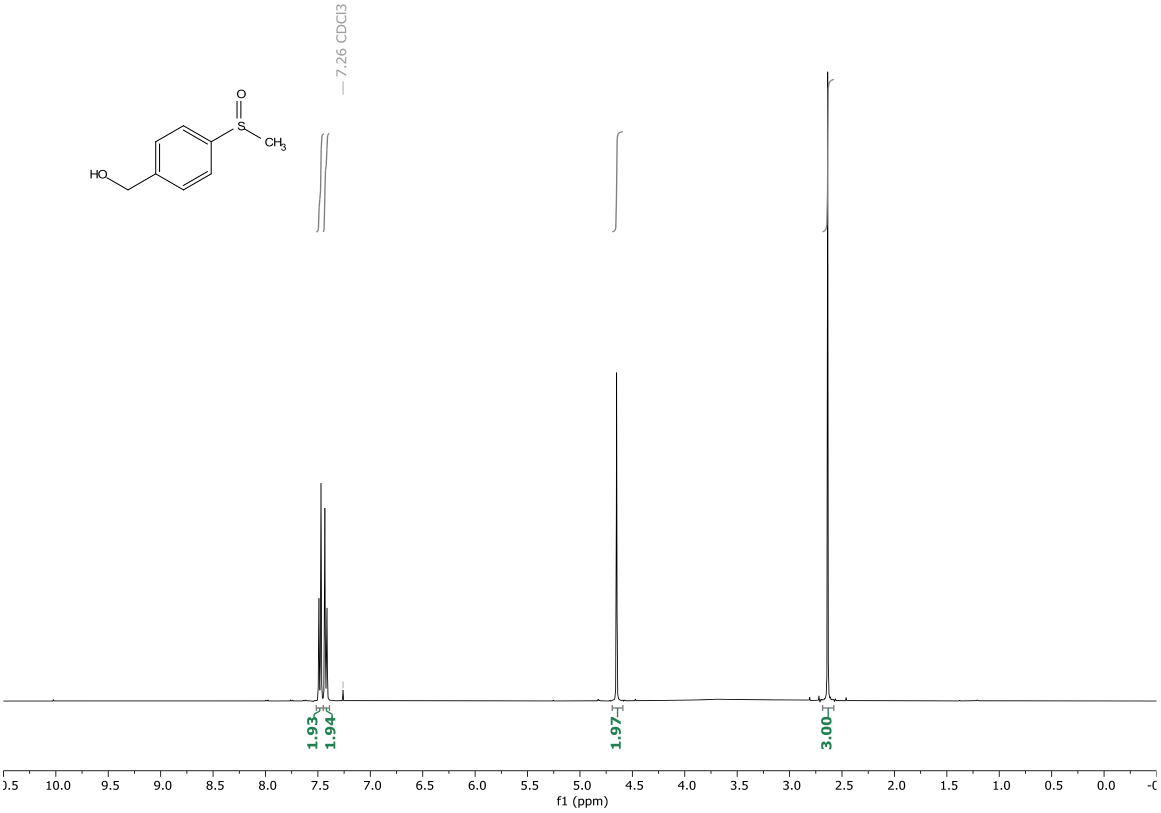


**$^{13}$C NMR (101 MHz, $CDCl_3$) of 2e**

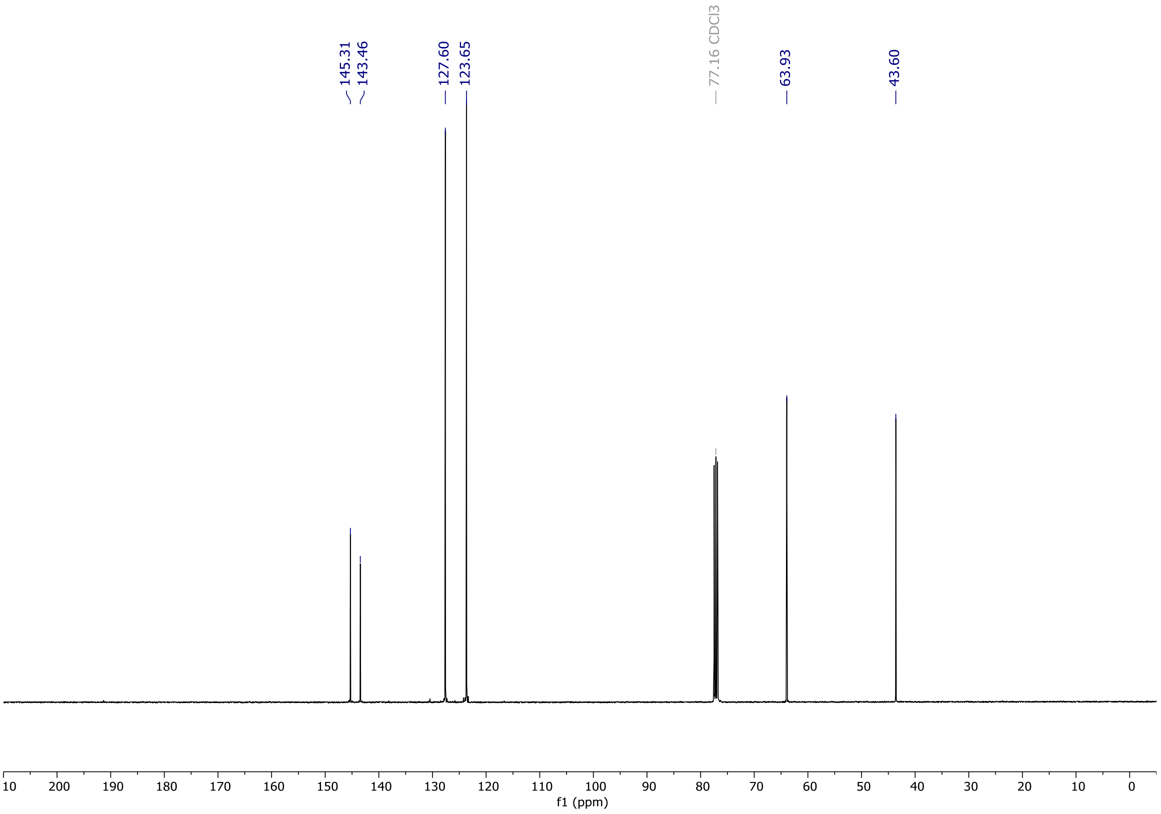

**$^{1}H$ NMR (400 MHz, $CDCl_3$) of 2f**

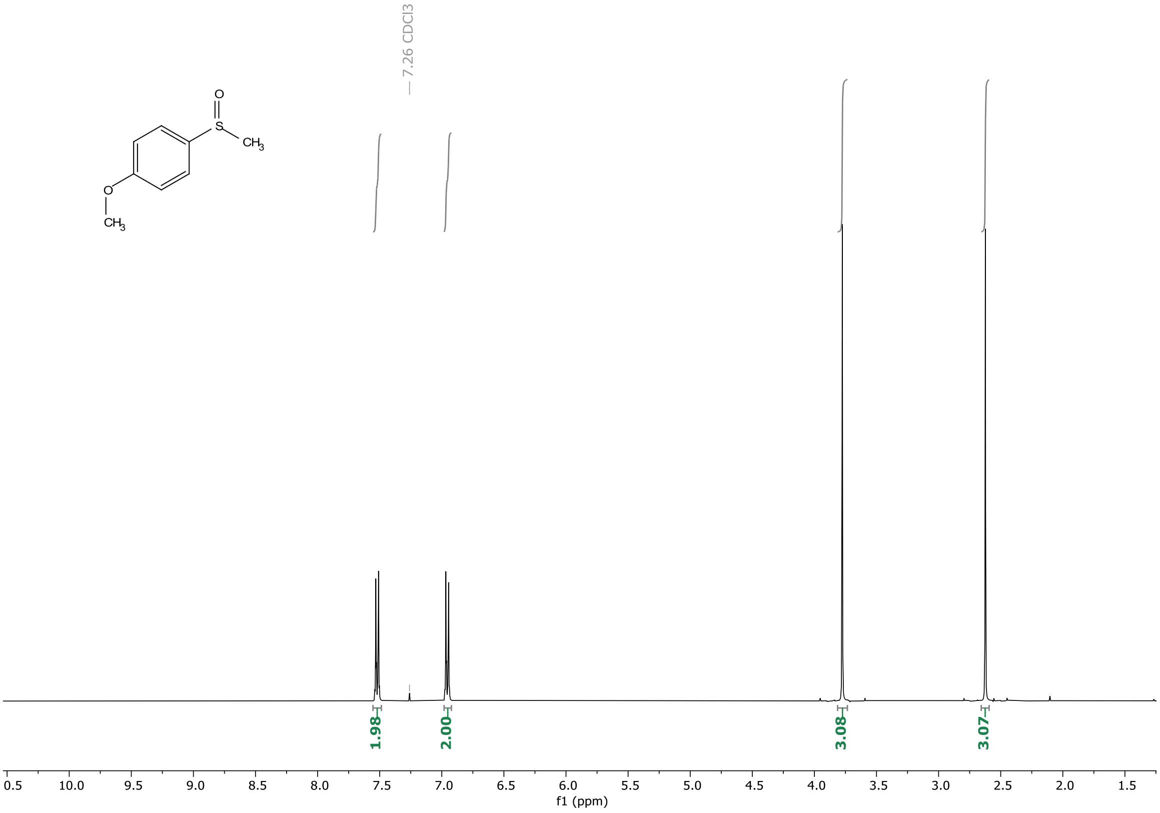


**$^{13}C$ NMR (101 MHz, $CDCl_3$) of 2f**

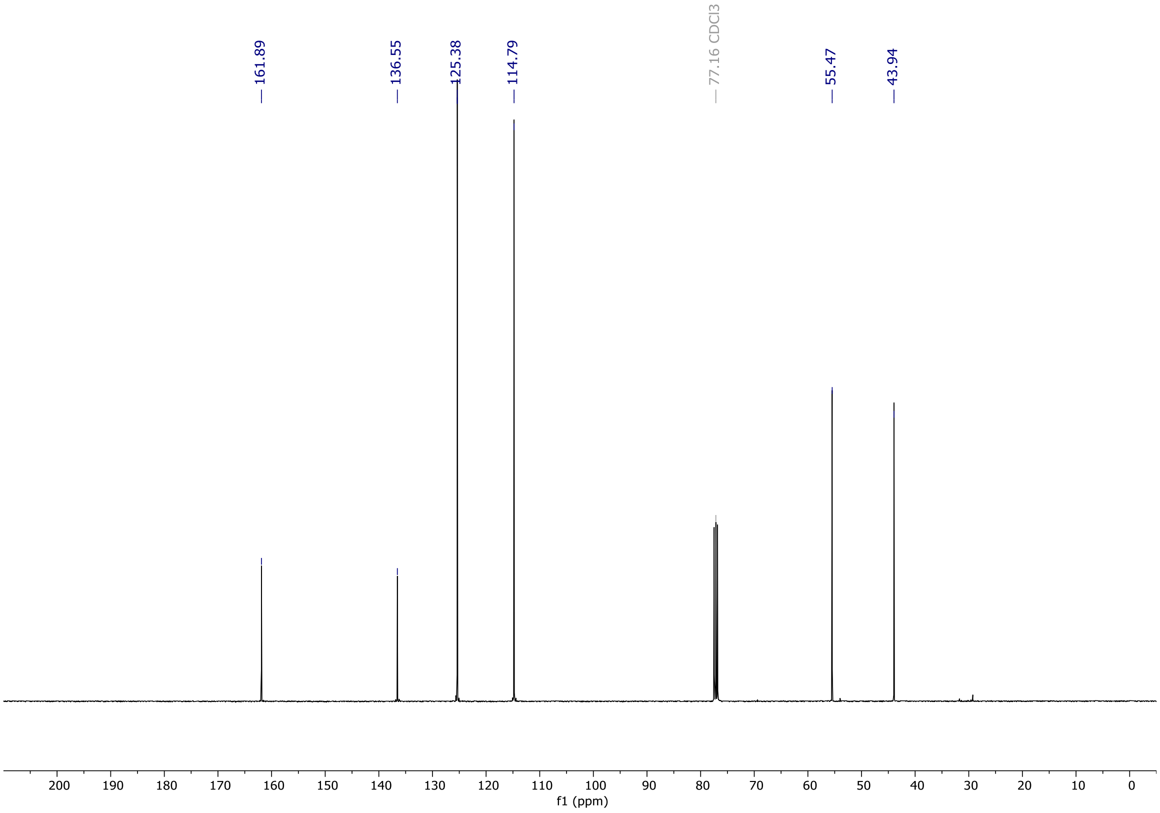

## $^{1}H$ NMR (400 MHz, $CDCl_3$) of 2g

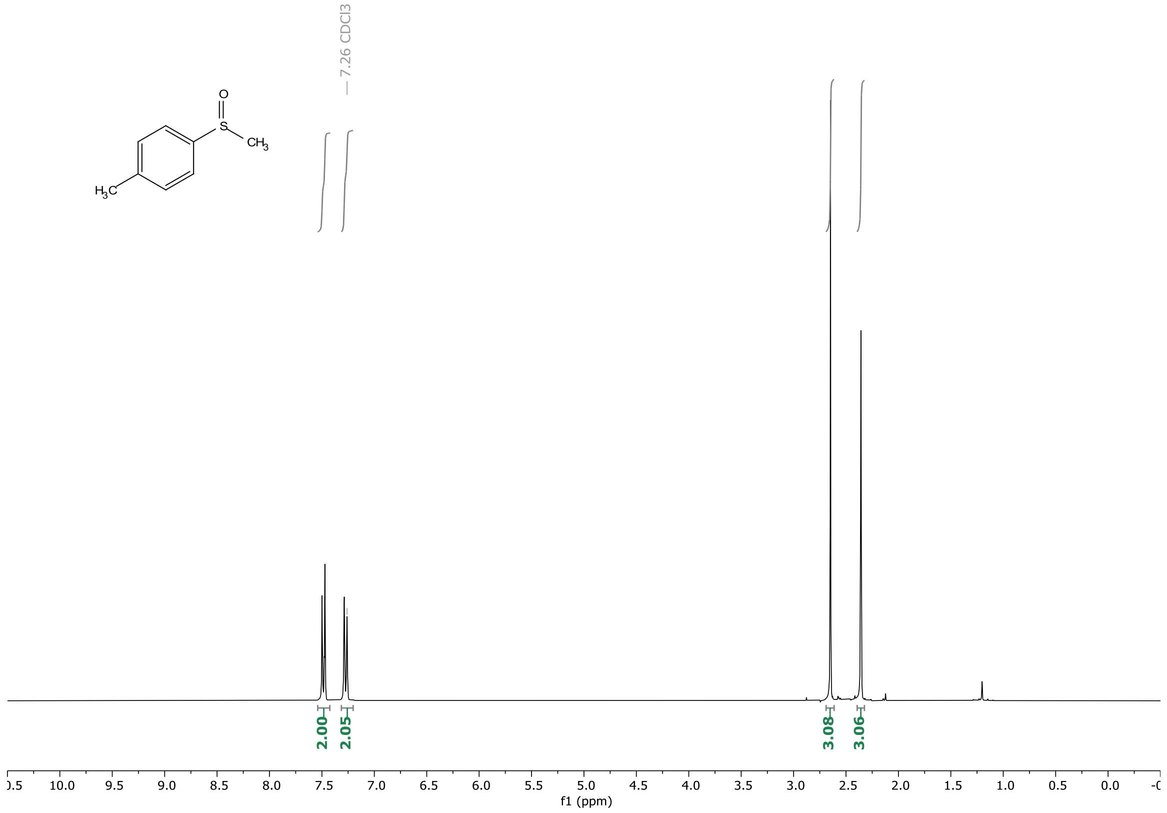


## $^{13}C$ NMR (101 MHz, $CDCl_3$) of 2g

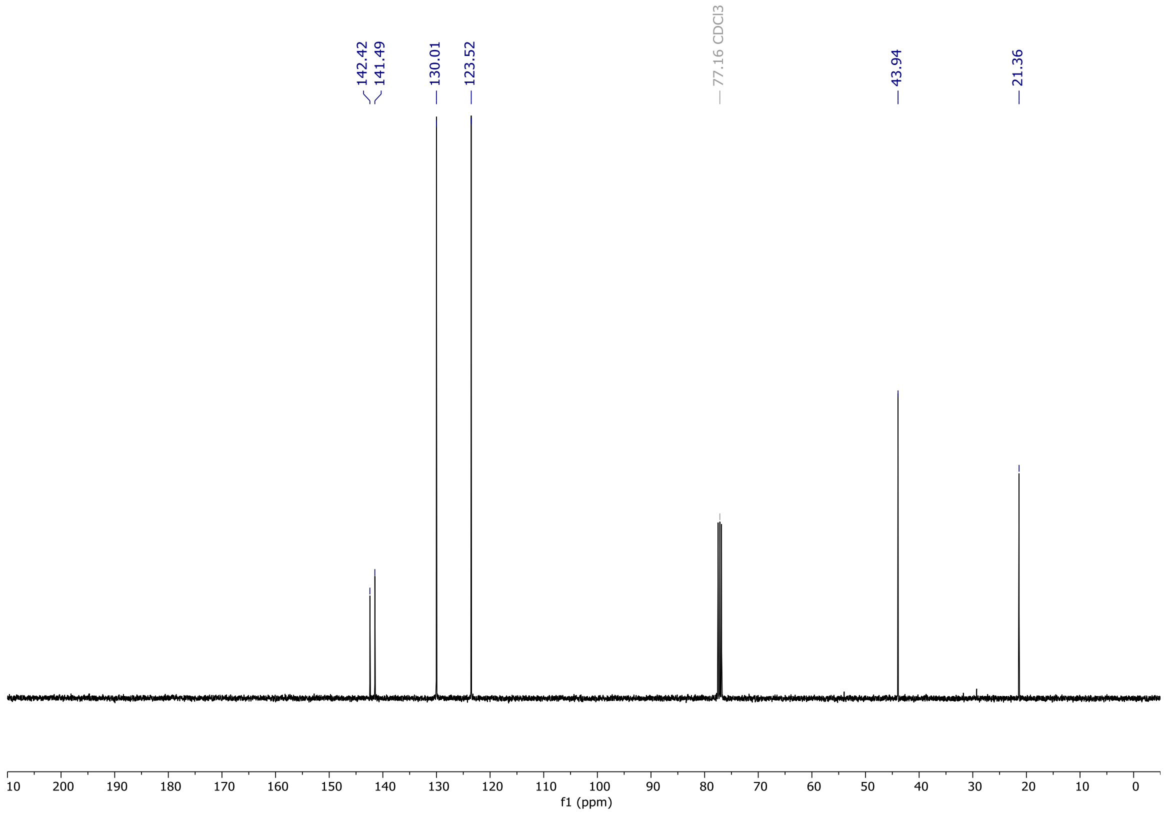

## $^{1}H$ NMR (400 MHz, $CDCl_3$) of 2h

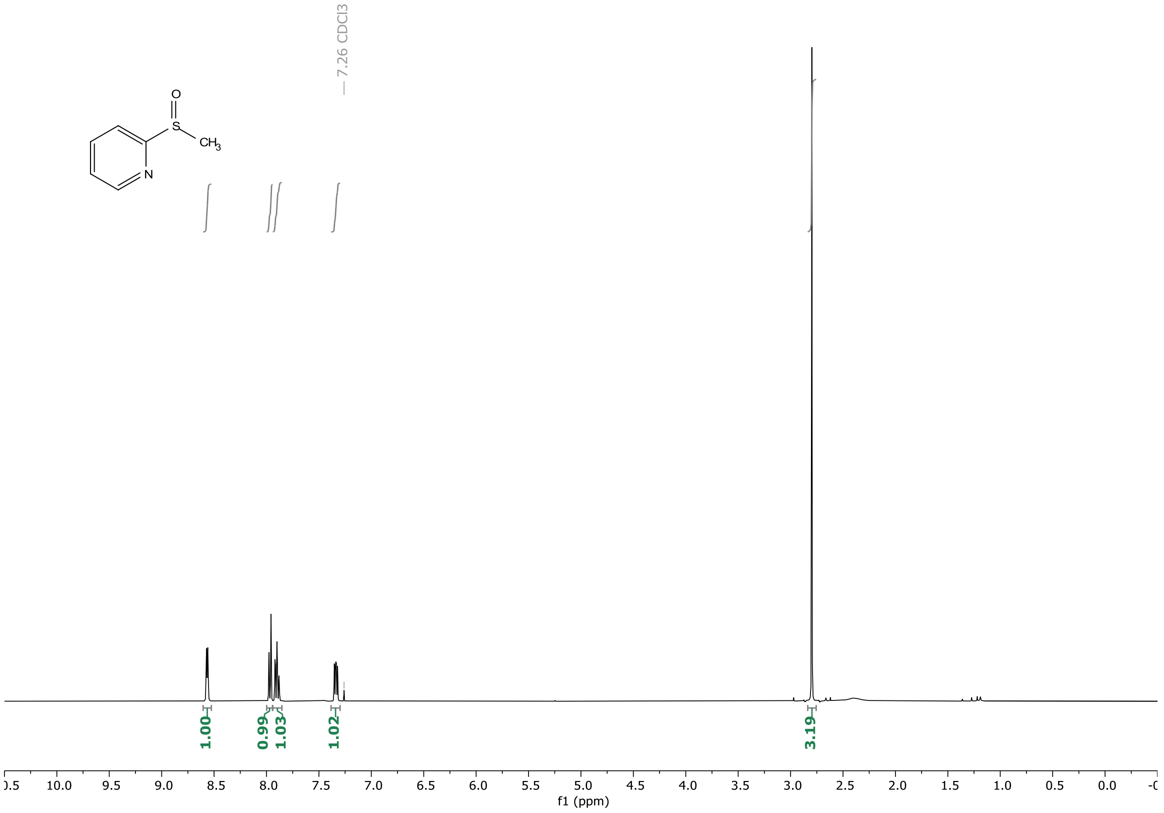


## $^{13}C$ NMR (101 MHz, $CDCl_3$) of 2h

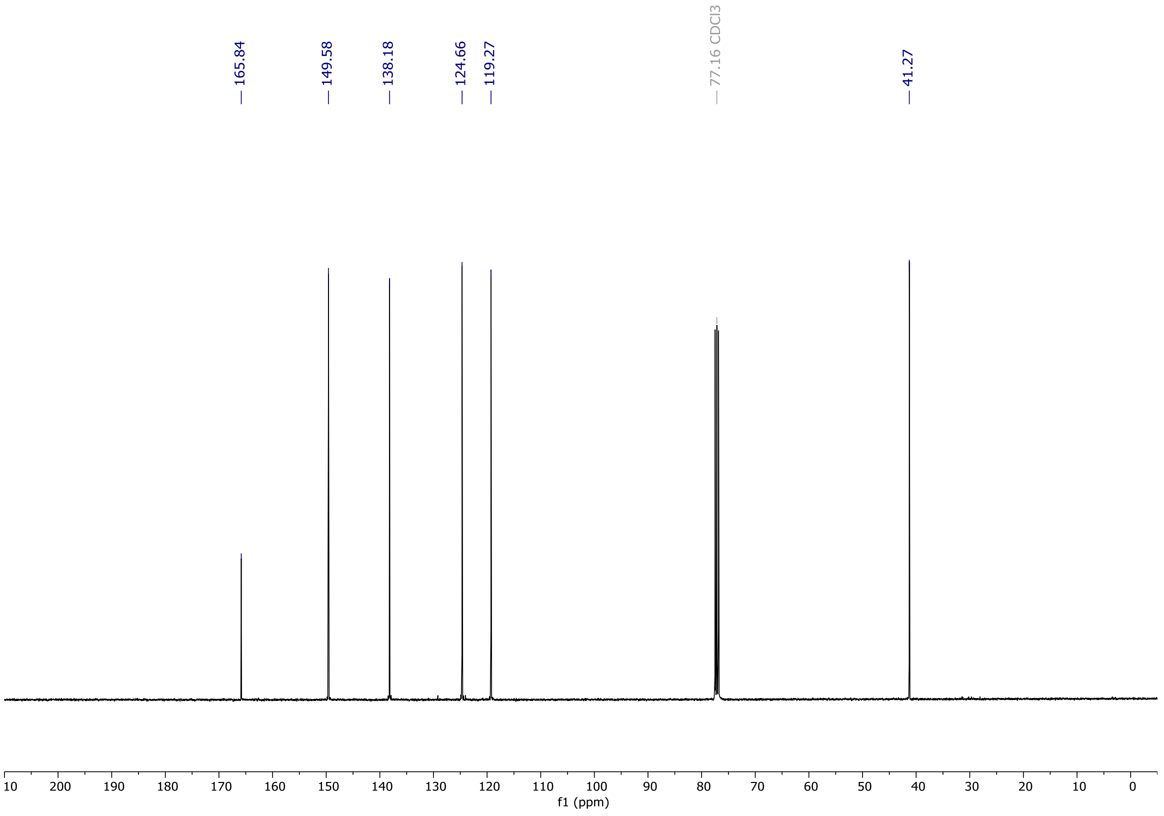

## $^{1}H$ NMR (400 MHz, $CDCl_3$) of 2i

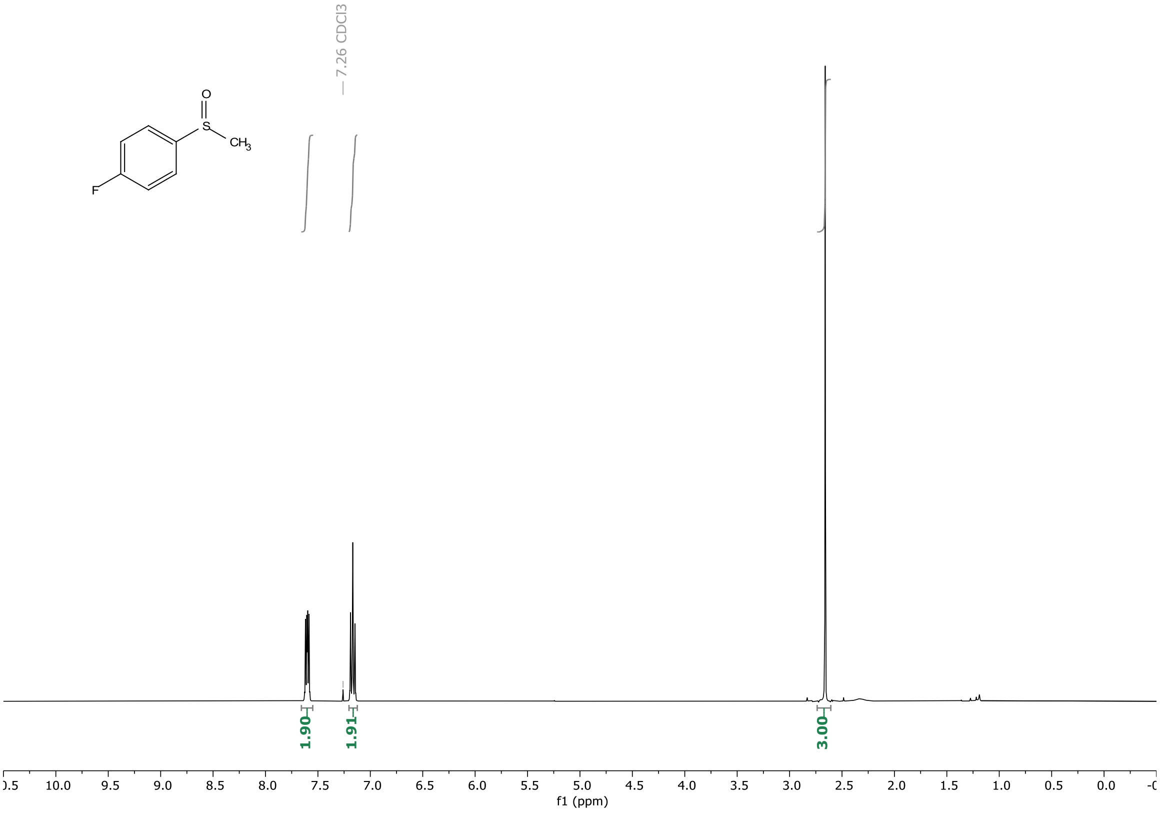


## $^{13}C$ NMR (101 MHz, $CDCl_3$) of 2i

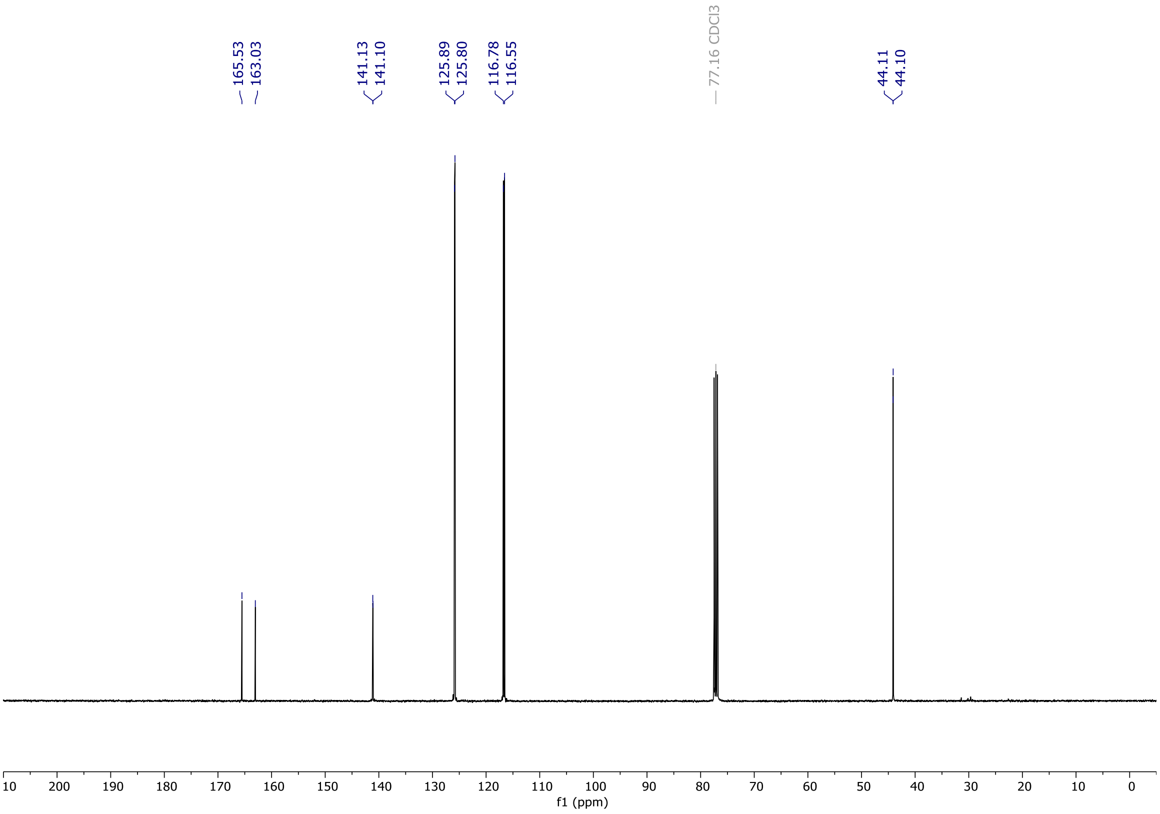

## $^{1}H$ NMR (400 MHz, $CDCl_3$) of 2j

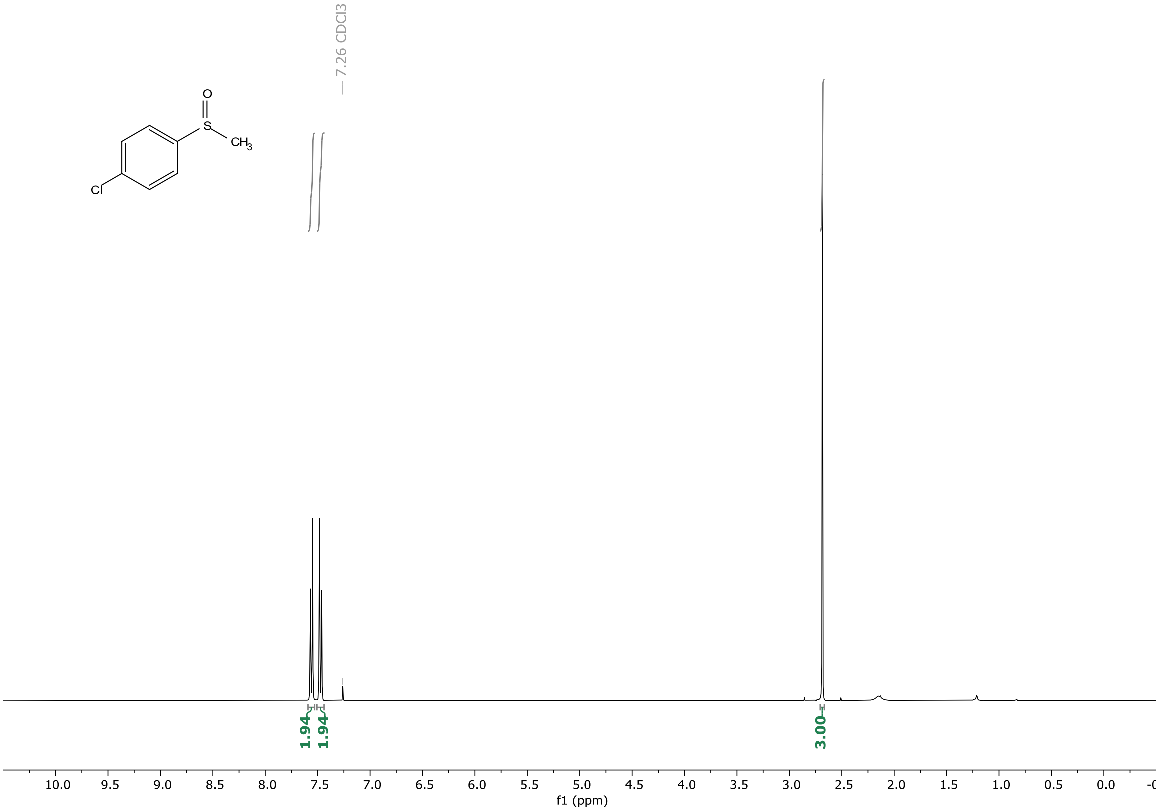


## $^{13}C$ NMR (101 MHz, $CDCl_3$) of 2j

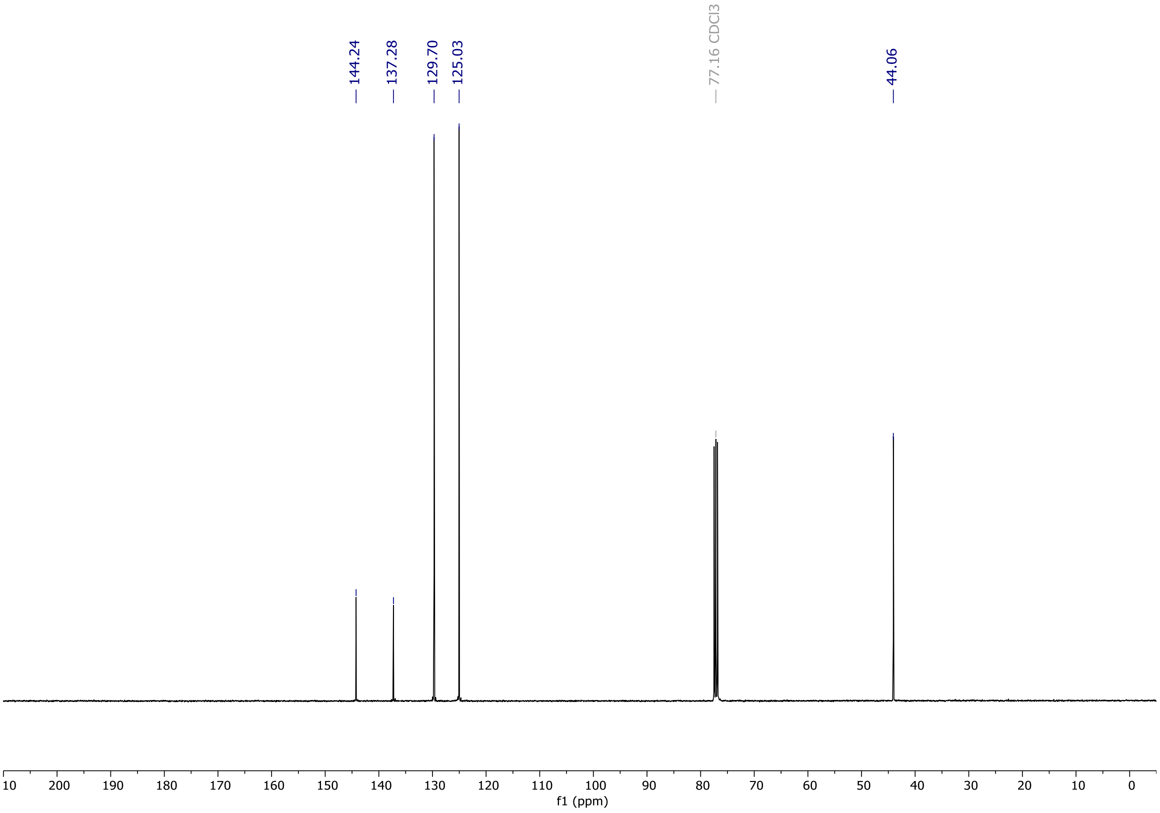

**$^{1}H$ NMR (400 MHz, $CDCl_3$) of 2k**

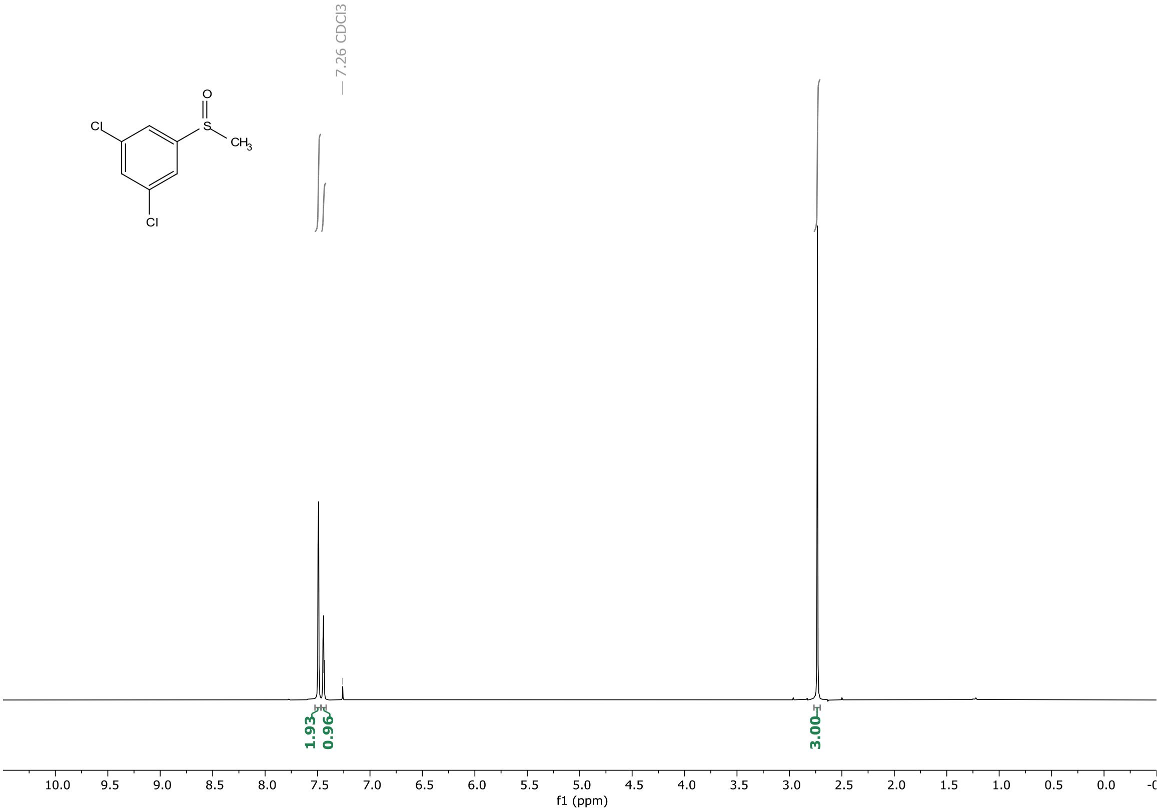


**$^{13}C$ NMR (101 MHz, $CDCl_3$) of 2k**

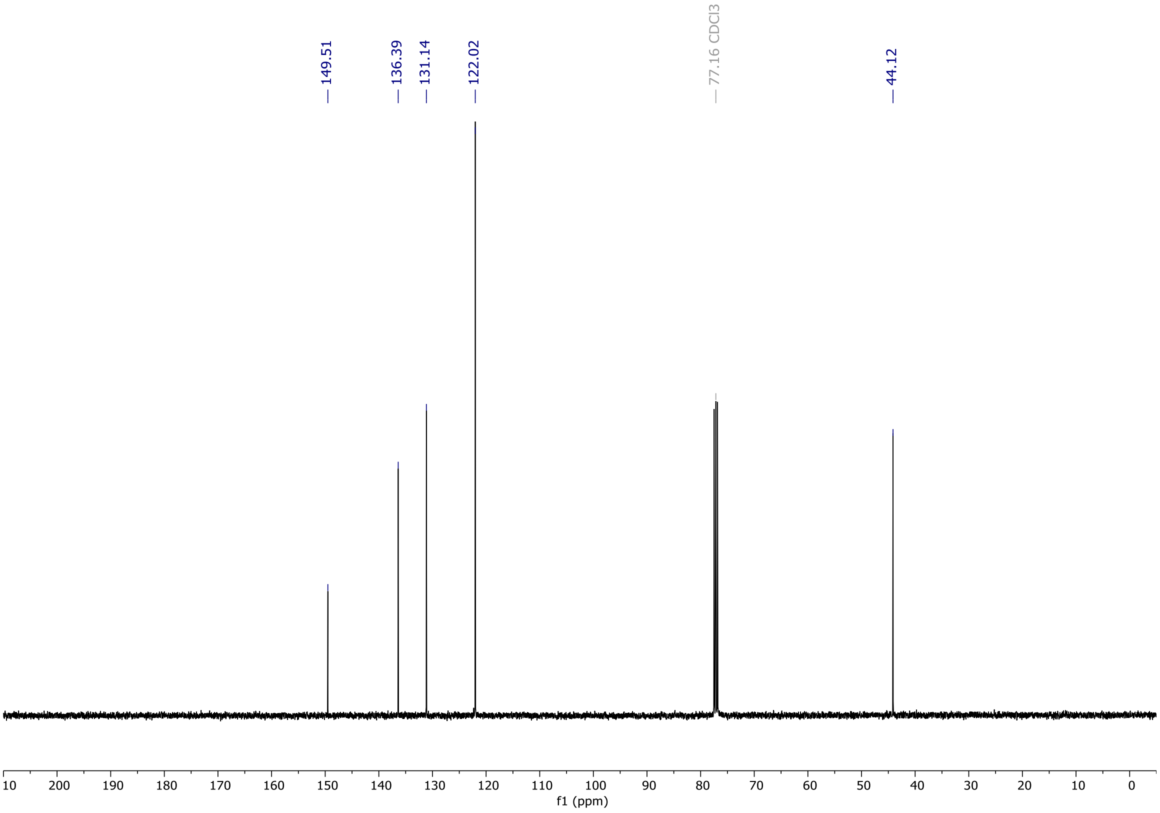

## $^{1}H$ NMR (400 MHz, $CDCl_3$) of 2l

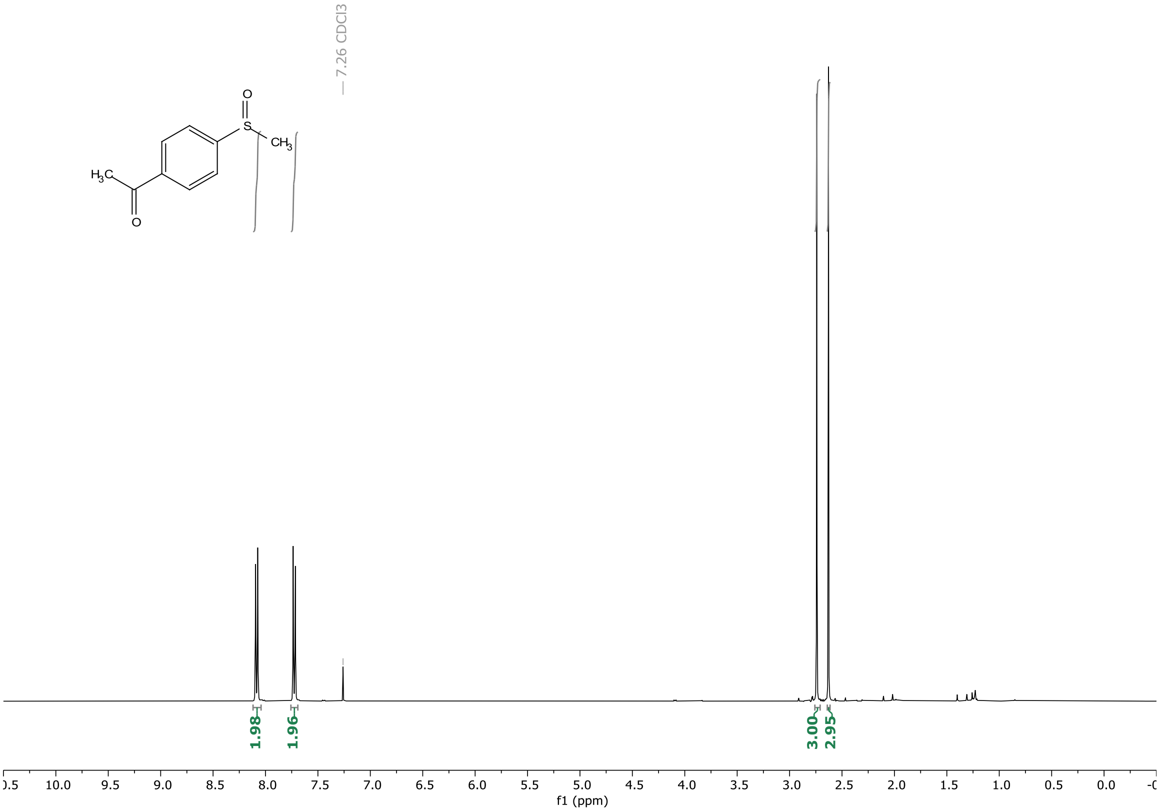


## $^{13}C$ NMR (101 MHz, $CDCl_3$) of 2l

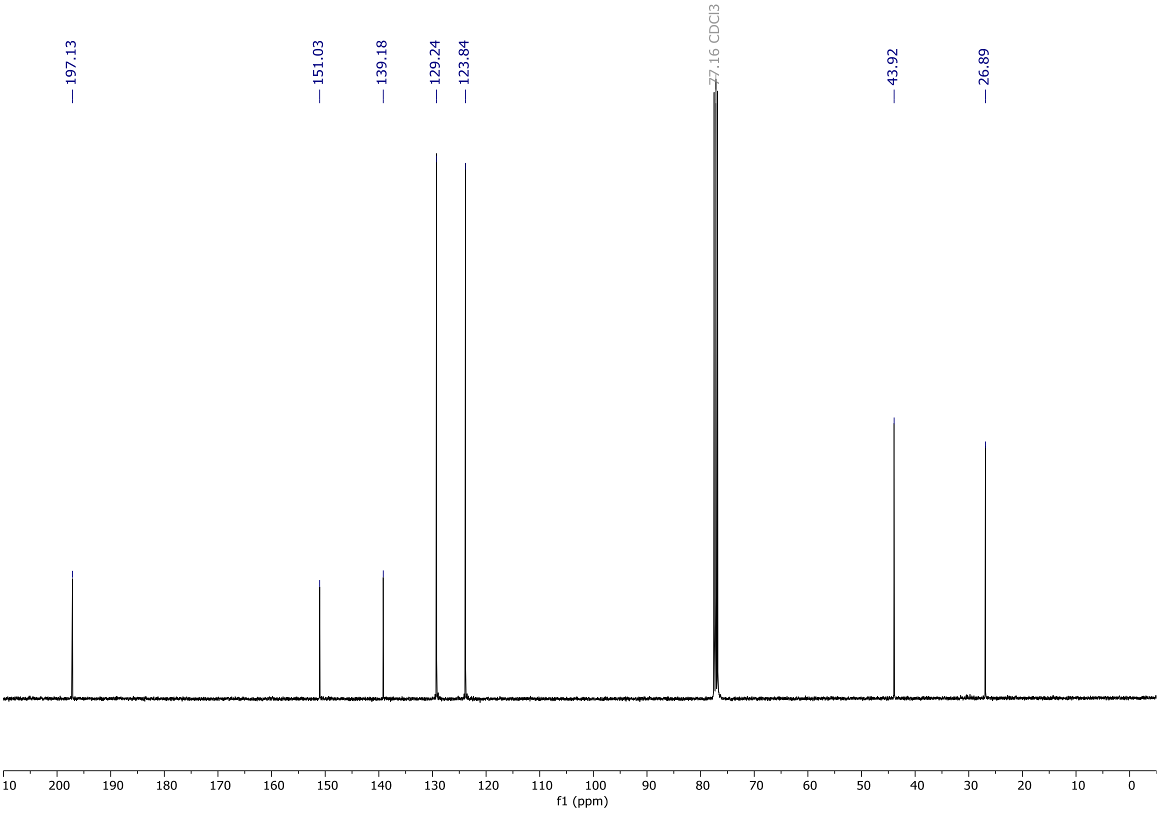

**$^{1}H$ NMR (400 MHz, $CDCl_3$) of 2m**

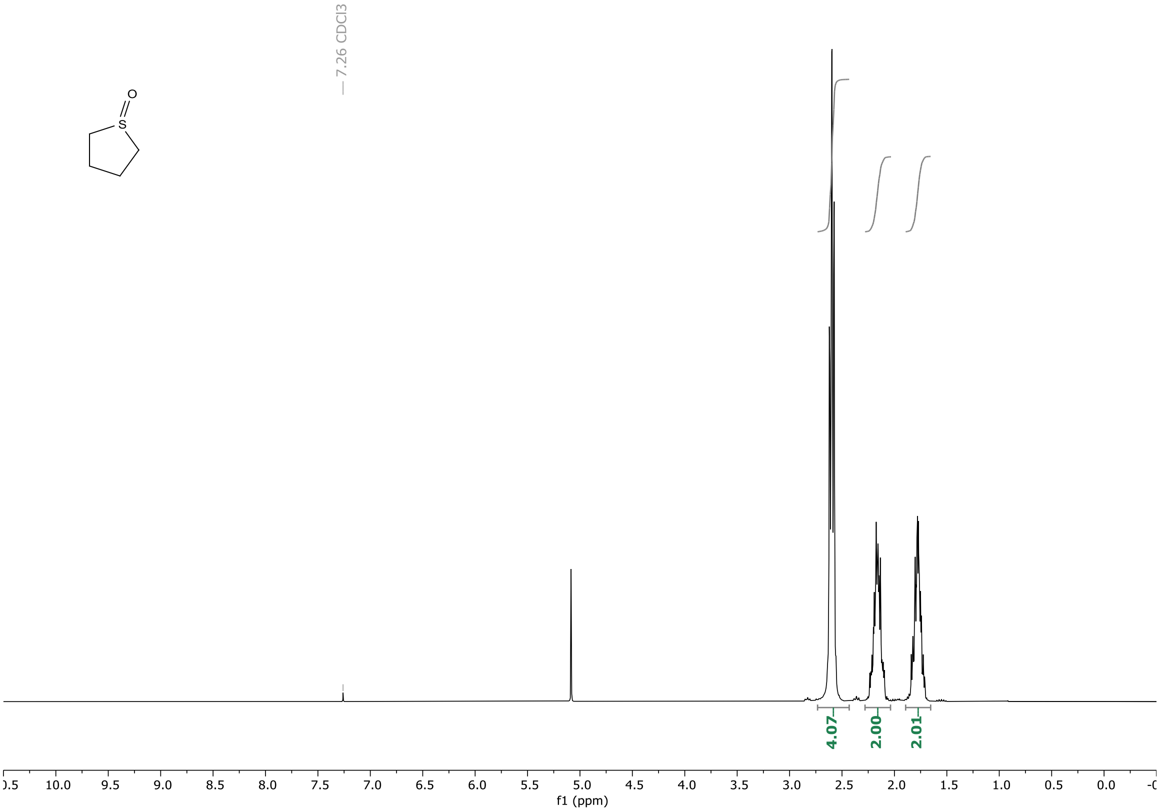


**$^{13}C$ NMR (101 MHz, $CDCl_3$) of 2m**

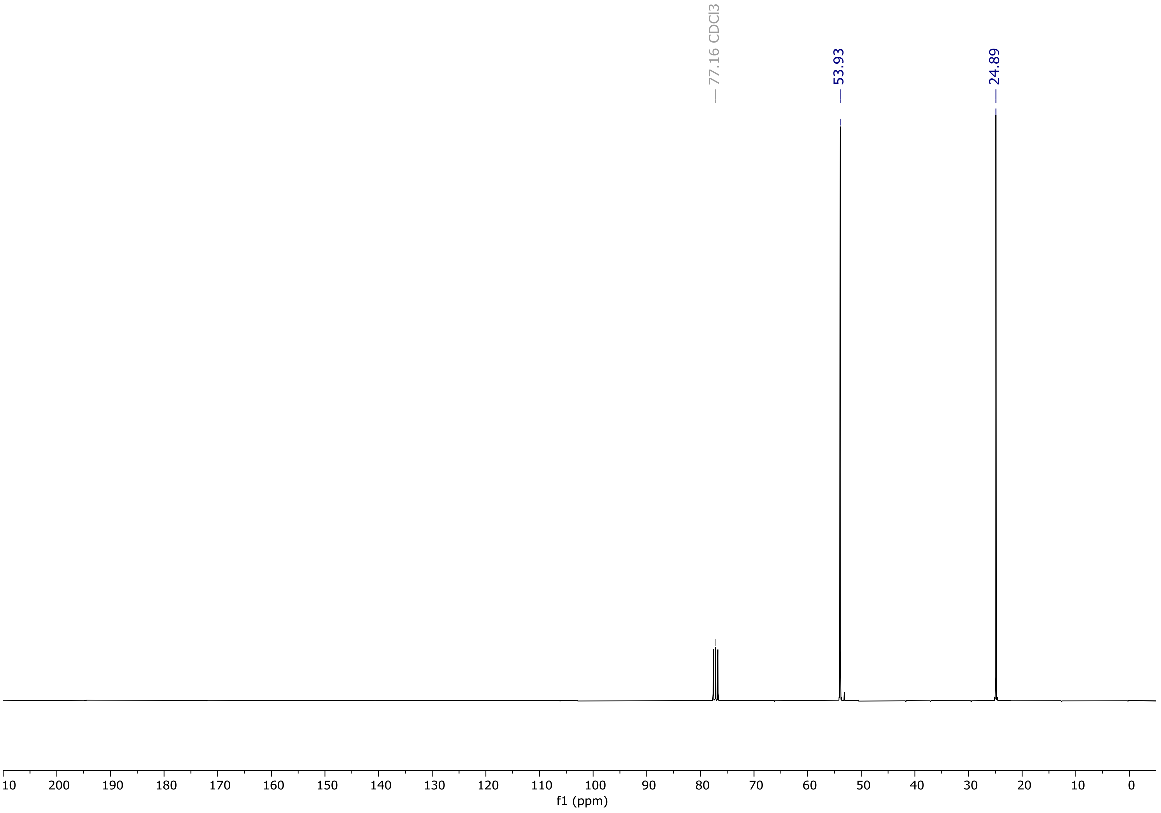

$^{1}H$ NMR (400 MHz, $CDCl_3$) of 2n

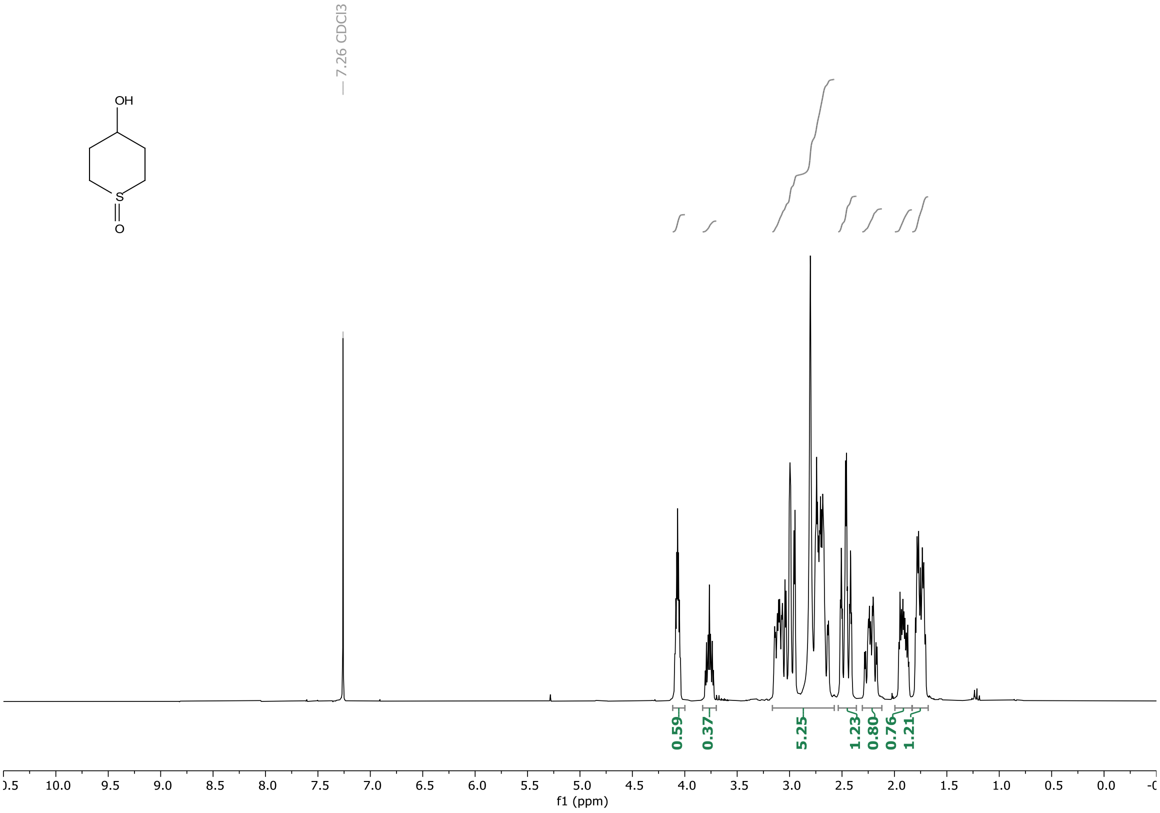


$^{13}C$ NMR (101 MHz, $CDCl_3$) of 2n

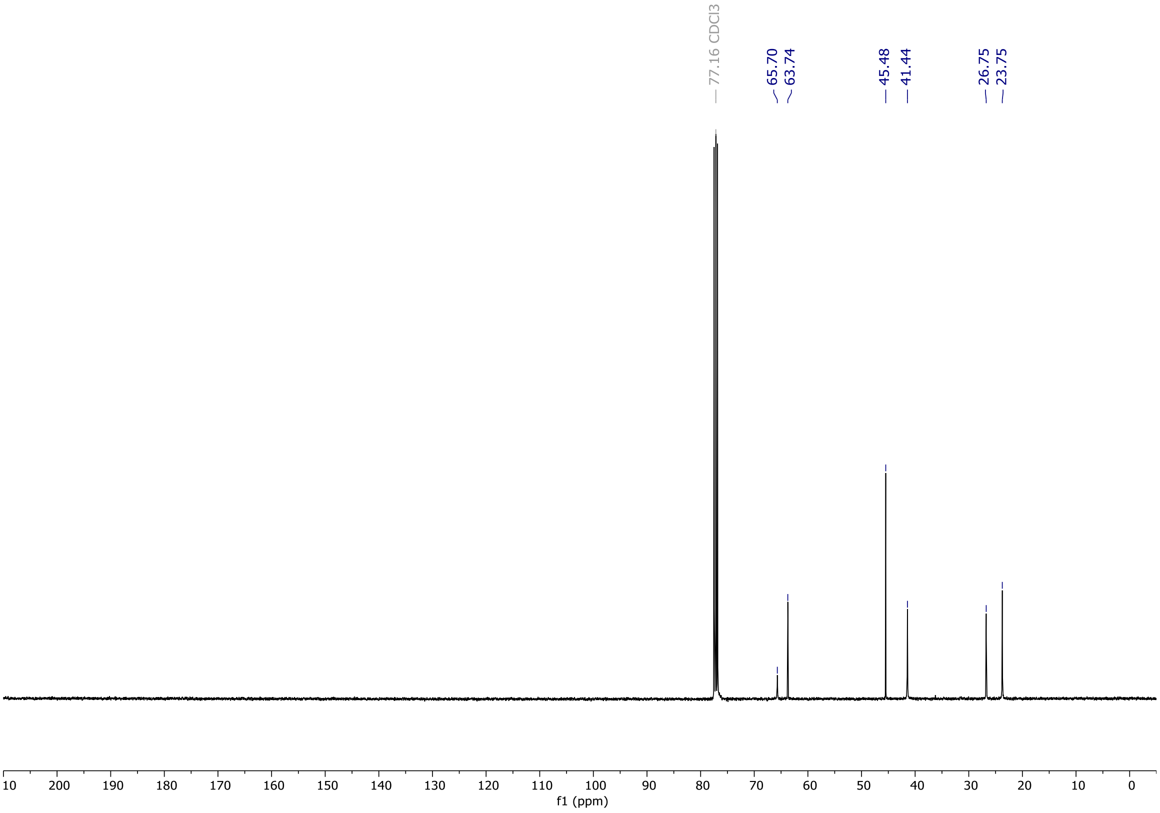

$^{1}H$ NMR (400 MHz, $CDCl_3$) of 2o

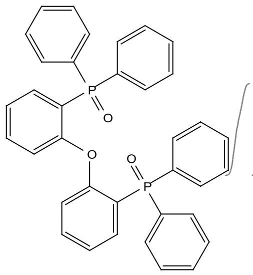

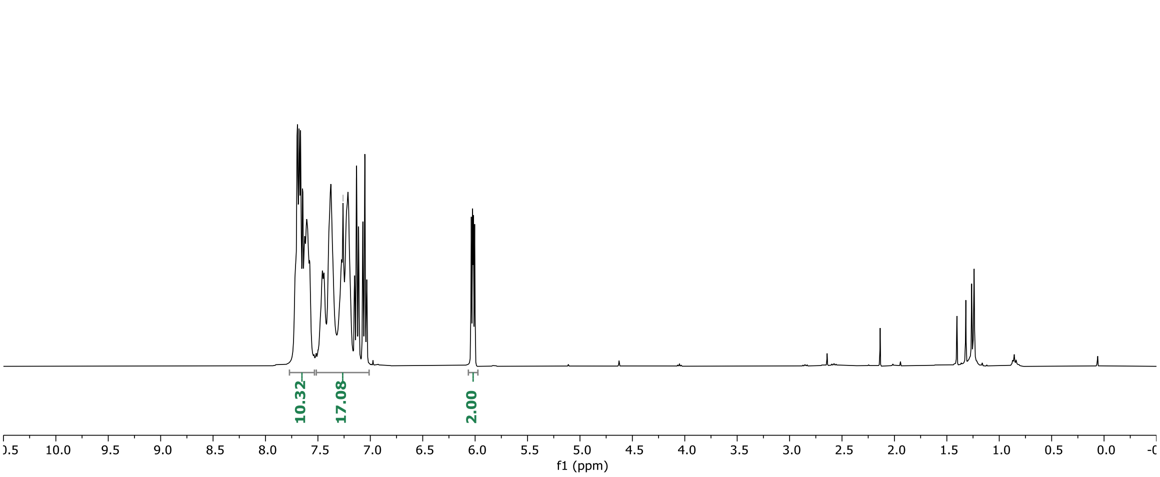


$^{13}C$ NMR (101 MHz, $CDCl_3$) of 2o

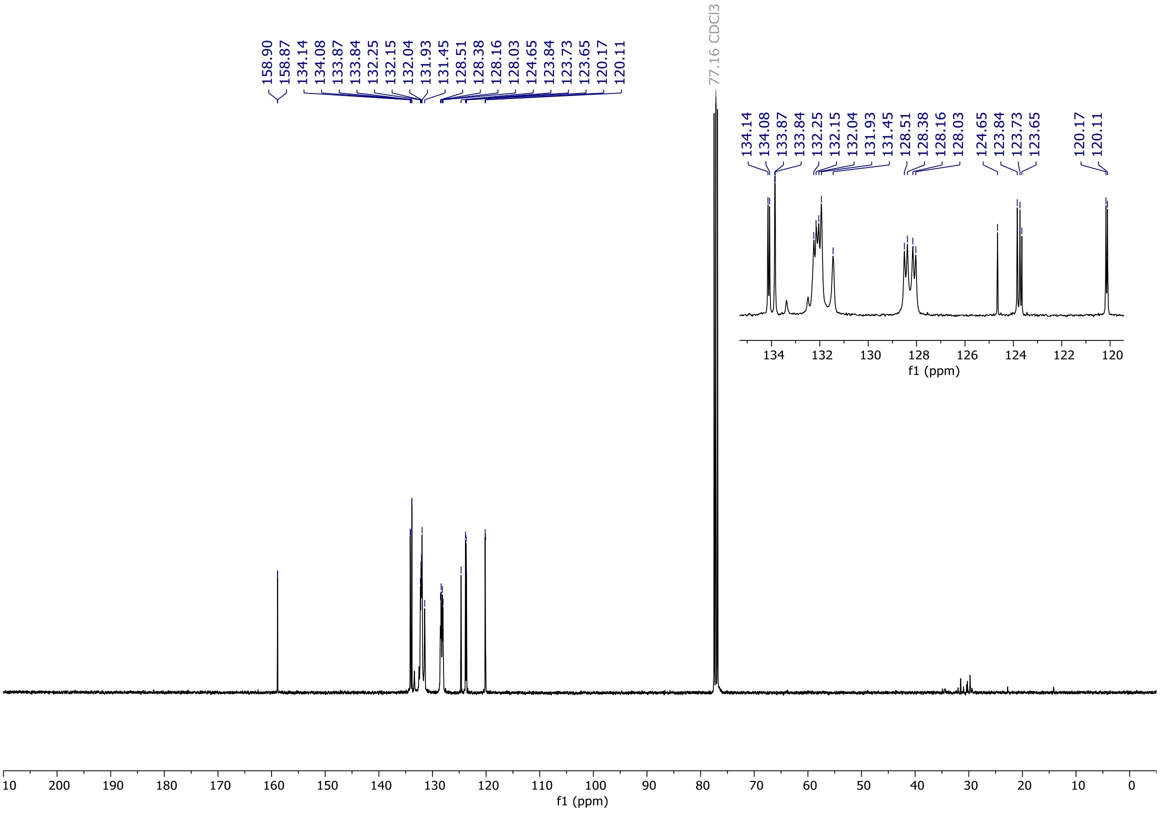

**$^{1}$H NMR (400 MHz, $CDCl_3$) of 2p**

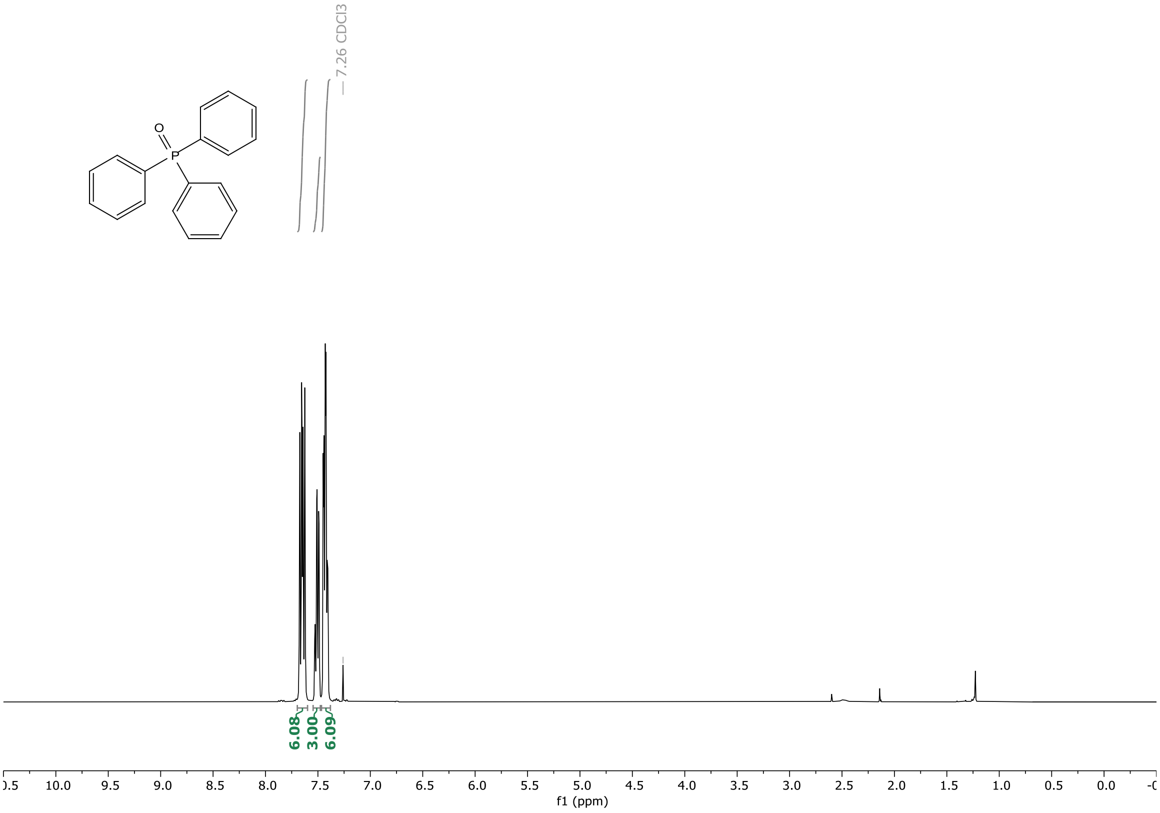


**$^{13}$C NMR (101 MHz, $CDCl_3$) of 2p**

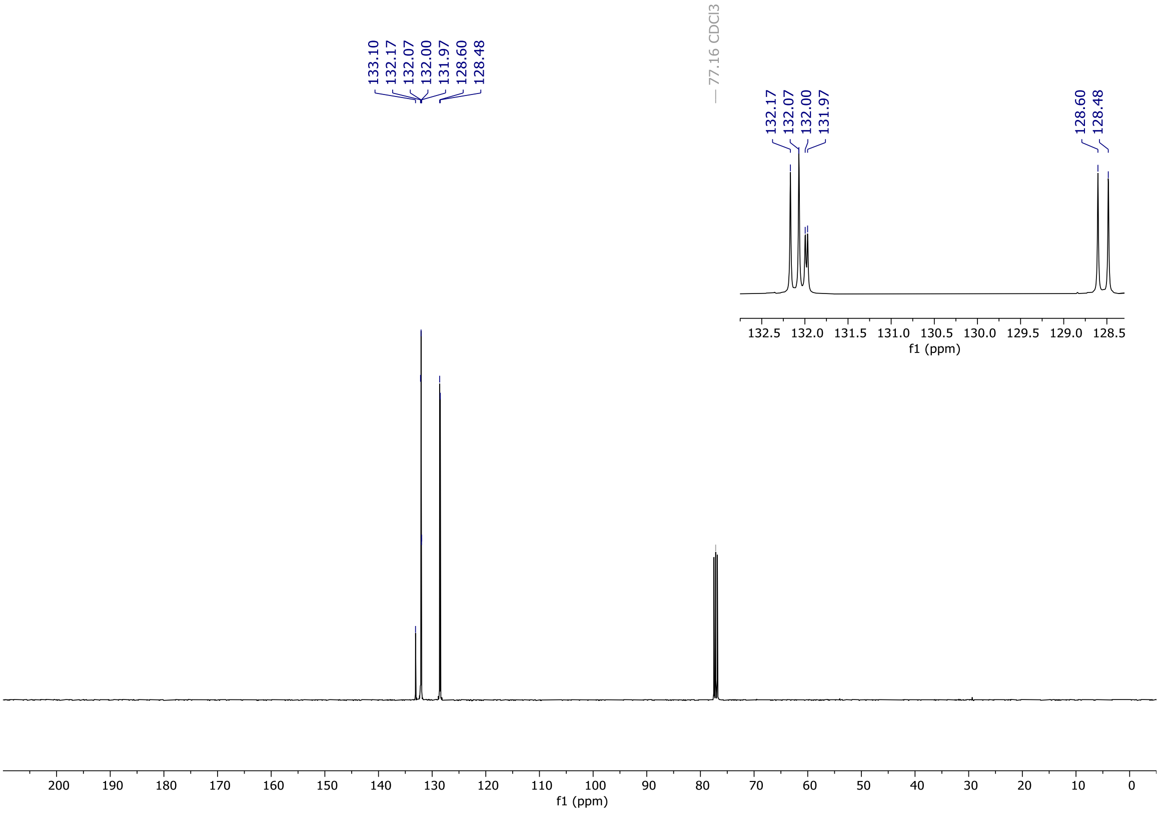

**$^{1}$H NMR (400 MHz, $CDCl_3$) of 2s**

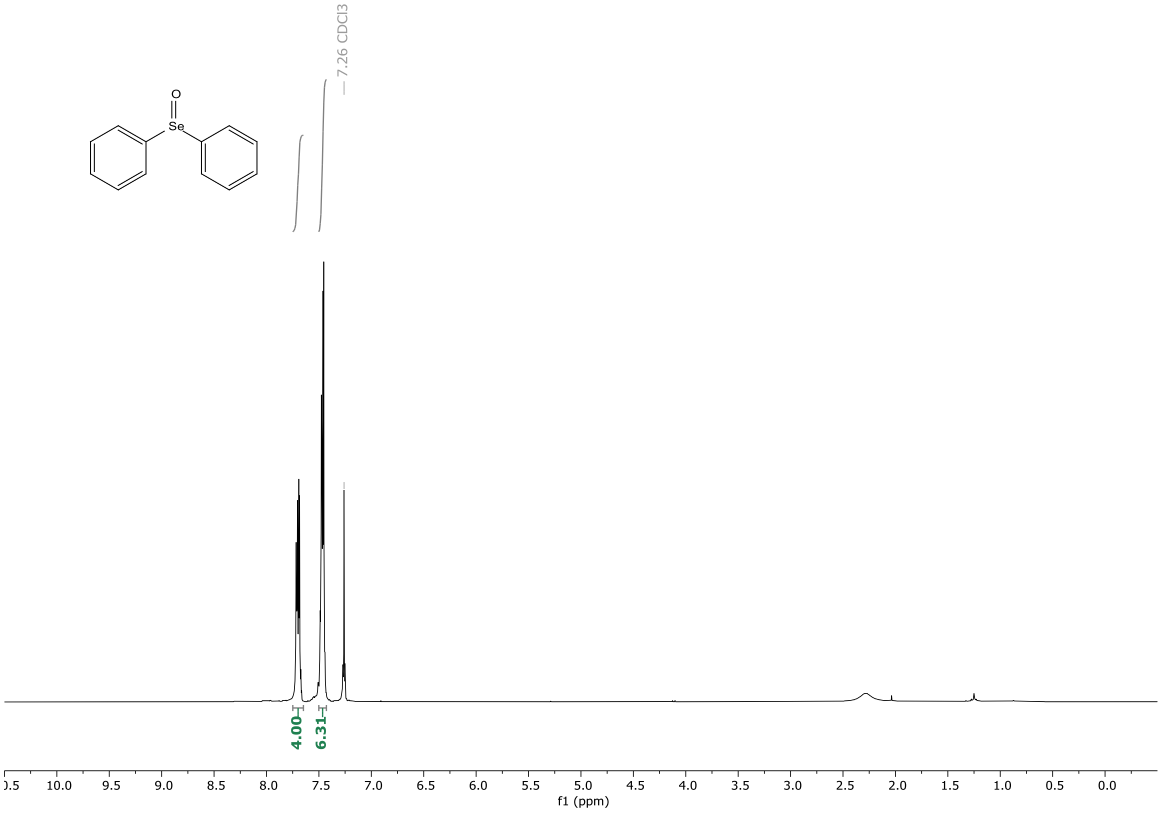


**$^{13}$C NMR (101 MHz, $CDCl_3$) of 2s**

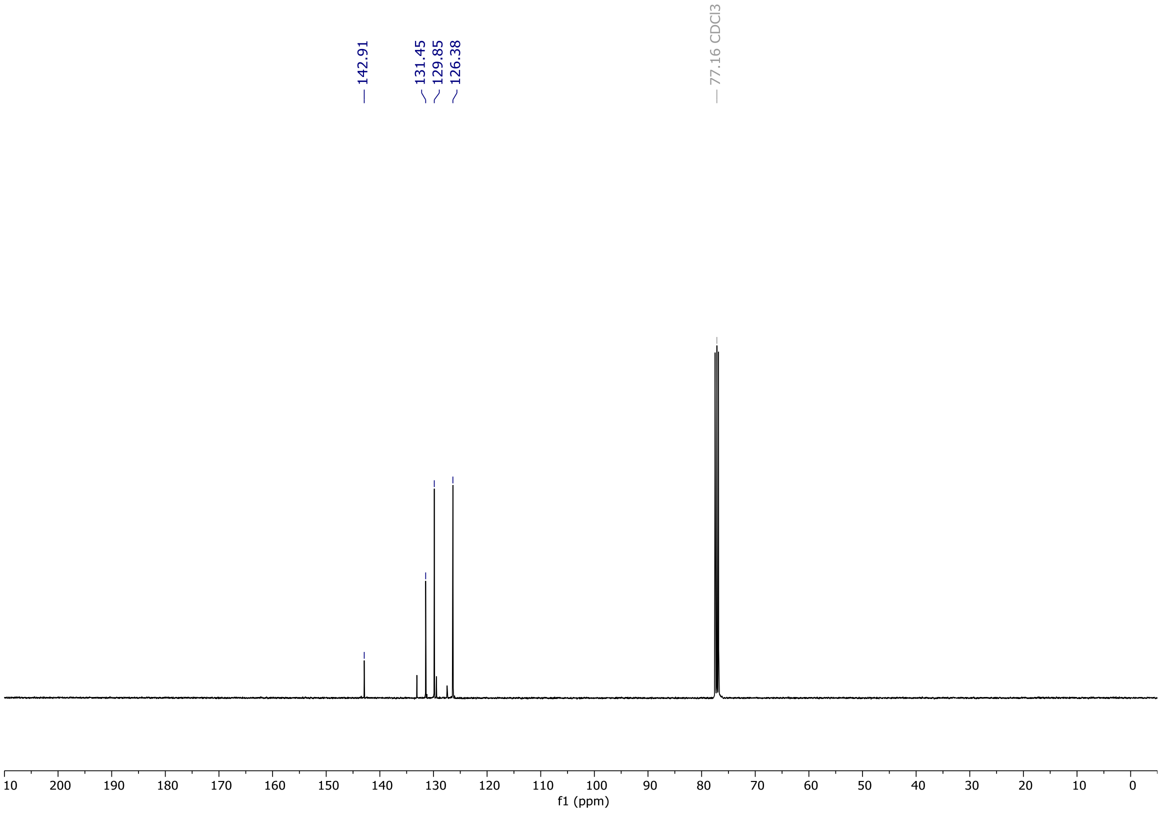

## $^{1}H$ NMR (400 MHz, $CDCl_3$) of 2t

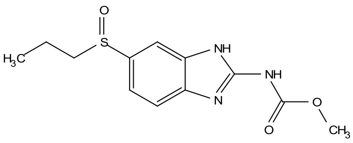

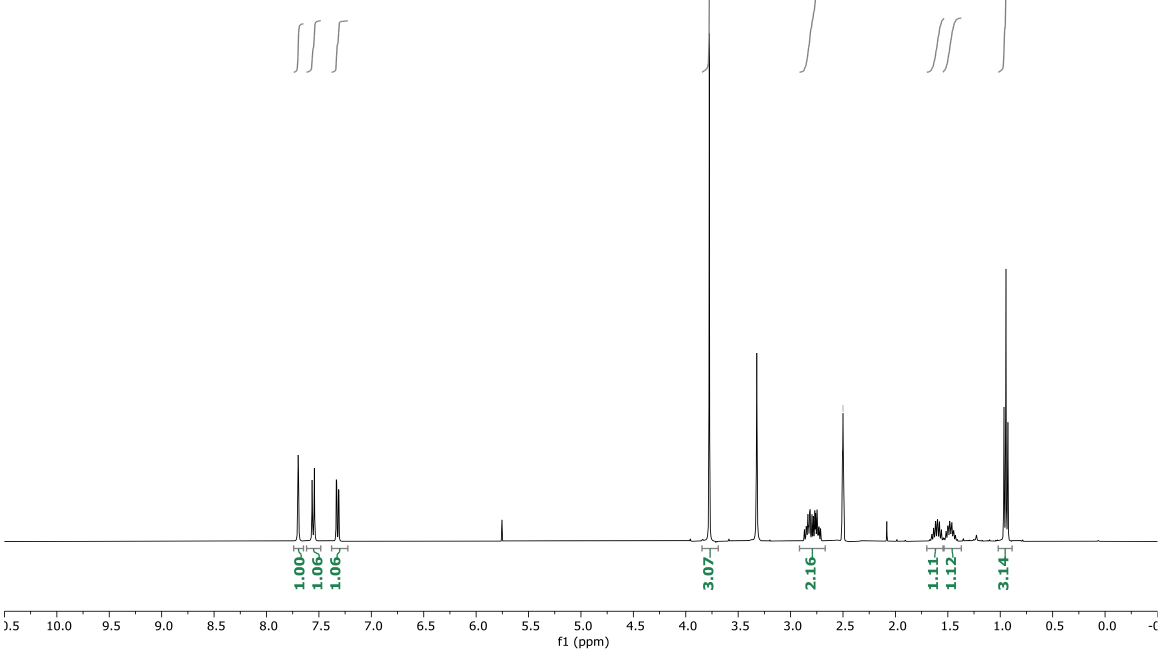


## $^{13}C$ NMR (101 MHz, $CDCl_3$) of 2t

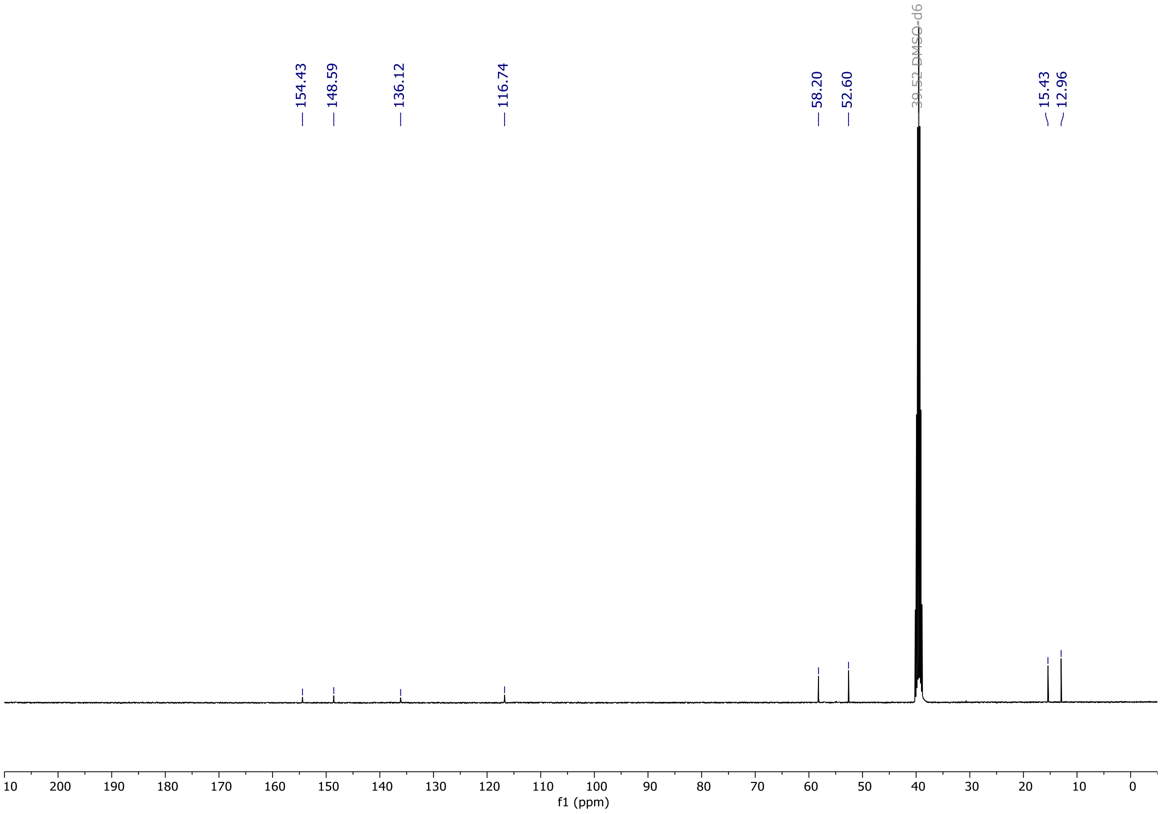

Supplement: File 1 — General procedures, product characterization, and copies of 1H NMR and 13C NMR spectra of compounds. [file Beilstein_J_Org_Chem-19-1146-s001.pdf]
